# Empowering chemical structures with biological insights for scalable phenotypic virtual screening

**DOI:** 10.1093/bioinformatics/btag517

**Published:** 2026-07-25

**Authors:** Xiaoqing Lian, Pengsen Ma, Tengfeng Ma, Zhonghao Ren, Xibao Cai, Zhixiang Cheng, Bosheng Song, He Wang, Xiang Pan, Yangyang Chen, Sisi Yuan, Chen Lin

**Affiliations:** State Key Laboratory of Chemo and Biosensing, College of Computer Science and Electronic Engineering, Hunan University, Changsha 410082, China; Yuelushan Laboratory, Changsha 410128, China; The Ministry of Education Key Laboratory of Fusion Computing of Supercomputing and Artificial Intelligence, Hunan University, Changsha 410082, China; State Key Laboratory of Chemo and Biosensing, College of Computer Science and Electronic Engineering, Hunan University, Changsha 410082, China; Yuelushan Laboratory, Changsha 410128, China; The Ministry of Education Key Laboratory of Fusion Computing of Supercomputing and Artificial Intelligence, Hunan University, Changsha 410082, China; State Key Laboratory of Chemo and Biosensing, College of Computer Science and Electronic Engineering, Hunan University, Changsha 410082, China; Yuelushan Laboratory, Changsha 410128, China; The Ministry of Education Key Laboratory of Fusion Computing of Supercomputing and Artificial Intelligence, Hunan University, Changsha 410082, China; State Key Laboratory of Chemo and Biosensing, College of Computer Science and Electronic Engineering, Hunan University, Changsha 410082, China; Yuelushan Laboratory, Changsha 410128, China; The Ministry of Education Key Laboratory of Fusion Computing of Supercomputing and Artificial Intelligence, Hunan University, Changsha 410082, China; State Key Laboratory of Chemo and Biosensing, College of Computer Science and Electronic Engineering, Hunan University, Changsha 410082, China; Yuelushan Laboratory, Changsha 410128, China; The Ministry of Education Key Laboratory of Fusion Computing of Supercomputing and Artificial Intelligence, Hunan University, Changsha 410082, China; State Key Laboratory of Chemo and Biosensing, College of Computer Science and Electronic Engineering, Hunan University, Changsha 410082, China; Yuelushan Laboratory, Changsha 410128, China; The Ministry of Education Key Laboratory of Fusion Computing of Supercomputing and Artificial Intelligence, Hunan University, Changsha 410082, China; State Key Laboratory of Chemo and Biosensing, College of Computer Science and Electronic Engineering, Hunan University, Changsha 410082, China; Yuelushan Laboratory, Changsha 410128, China; The Ministry of Education Key Laboratory of Fusion Computing of Supercomputing and Artificial Intelligence, Hunan University, Changsha 410082, China; School of Artificial Intelligence and Computer Science, Jiangnan University, Wuxi 214122, China; School of Artificial Intelligence and Computer Science, Jiangnan University, Wuxi 214122, China; Department of Computer Science, University of Tsukuba, Tsukuba, Ibaraki, Japan; School of Chinese Medicine, Hong Kong Baptist University, Kowloon, Hong Kong SAR 999077, China; School of Informatics, National Institute for Data Science in Health and Medicine, Xiamen University, Xiamen 361000, China

## Abstract

**Motivation:**

The scalable identification of bioactive compounds is essential for contemporary drug discovery. This process faces a key trade-off: structural screening offers scalability but lacks biological context, whereas high-content phenotypic profiling provides deep biological insights but is resource-intensive. The primary challenge is to extract robust biological signals from noisy data and encode them into representations that do not require biological data at inference.

**Results:**

This study presents DECODE (DEcomposing Cellular Observations of Drug Effects), a framework that bridges this gap by empowering chemical representations with intrinsic biological semantics to enable structure-based in silico biological profiling. DECODE leverages limited paired transcriptomic and morphological data as supervisory signals during training, enabling the extraction of a measurement-invariant biological fingerprint from chemical structures and explicit filtering of modality-specific variation. Across held-out retrieval, scaffold-split and UMAP-clustering virtual-screening benchmarks, DECODE improves functional retrieval and early active-compound prioritization over baselines.

**Availability and implementation:**

The codes and datasets of DECODE are available at https://github.com/lian-xiao/DECODE.

## 1 Introduction

Early-stage drug discovery aims to identify novel therapeutic candidates within a nearly infinite chemical space (Goh *et al.* 2017). Success depends not only on molecular binding affinity but also on the ability of compounds to induce desired functional perturbations in complex biological systems (Swinney 2013). Consequently, phenotypic evaluation of large compound libraries is essential for prioritizing high-quality leads and reducing the high attrition rates typically seen in clinical development (Hughes *et al.* 2021).

Despite its importance, a fundamental trade-off remains between screening throughput and biological depth (Gryniukova *et al.* 2023). While chemical structures are readily available for billions of molecules, these static representations often fail to capture the dynamic functional landscapes of cellular responses (Ross *et al.* 2022, Masarone *et al.* 2025). In contrast, high-content phenotypic profiling (such as transcriptomics and morphological imaging)rovides direct readouts of drug action (Subramanian *et al.* 2017, Chandrasekaran *et al.* 2021). However, generating these profiles requires expensive wet-lab experiments, making it economically impractical to profile the vast chemical libraries accessible via virtual screening (Haghighi *et al.* 2022). A practical way to bridge this gap is to use existing phenotypic profiles as supervision, so that structure-based models can learn molecular representations that better reflect drug-induced biological responses.

Two challenges make this non-trivial. First, chemical similarity does not always imply functional similarity: structurally different compounds can elicit similar cellular responses, whereas small structural changes can cause large shifts in activity. Thus, useful representations should capture biological relationships beyond molecular similarity alone. Second, transcriptomic and morphological profiles are noisy and affected by batch effects and assay-specific artifacts (Schäfer *et al.* 2024). Directly fitting these profiles may transfer technical noise into molecular representations (He *et al.* 2021). The key problem, therefore, is not simply to reconstruct biological profiles from structure, but to extract shared response-related information that improves downstream screening.

To address this problem, we introduce DECODE (DEcomposing Cellular Observations of Drug Effects), a framework for learning biologically informed molecular representations. DECODE uses paired transcriptomic (gene expression measurements) and morphological (cell shape and structure) profiles as privileged information during training (Vapnik and Vashist 2009, Bray *et al.* 2016, Subramanian *et al.* 2017), while not requiring these profiles as inputs during inference. Compared with conventional structure-based molecular encoders, DECODE guides chemical representations using cellular response profiles. Beyond standard multimodal alignment or response-prediction settings, it further separates response-shared signals from modality-specific variation. This backbone-flexible design allows different molecular encoders to be enhanced with biological-profile supervision without requiring newly measured biological profiles at inference (Ross *et al.* 2022, Wu *et al.* 2022, Moshkov *et al.* 2023, Sanchez-Fernandez *et al.* 2023, Xiang *et al.* 2024, Zheng *et al.* 2024).

We evaluate DECODE in held-out functional retrieval, modality-flexible MoA prediction, and downstream virtual-screening tasks. For virtual screening, we use chemically informed splits and early-recognition metrics, including BEDROC and enrichment factors (Bemis and Murcko 1996, Truchon and Bayly 2007, Guo *et al.* 2025). We further compare with stronger molecular and multimodal baselines to test whether the gains come from biological-profile supervision rather than backbone strength or predictor capacity. These results support DECODE as a scalable framework for biologically informed virtual screening.

## 2 Methods

### 2.1 Problem formulation: learning with privileged biological context

**Figure 1:**
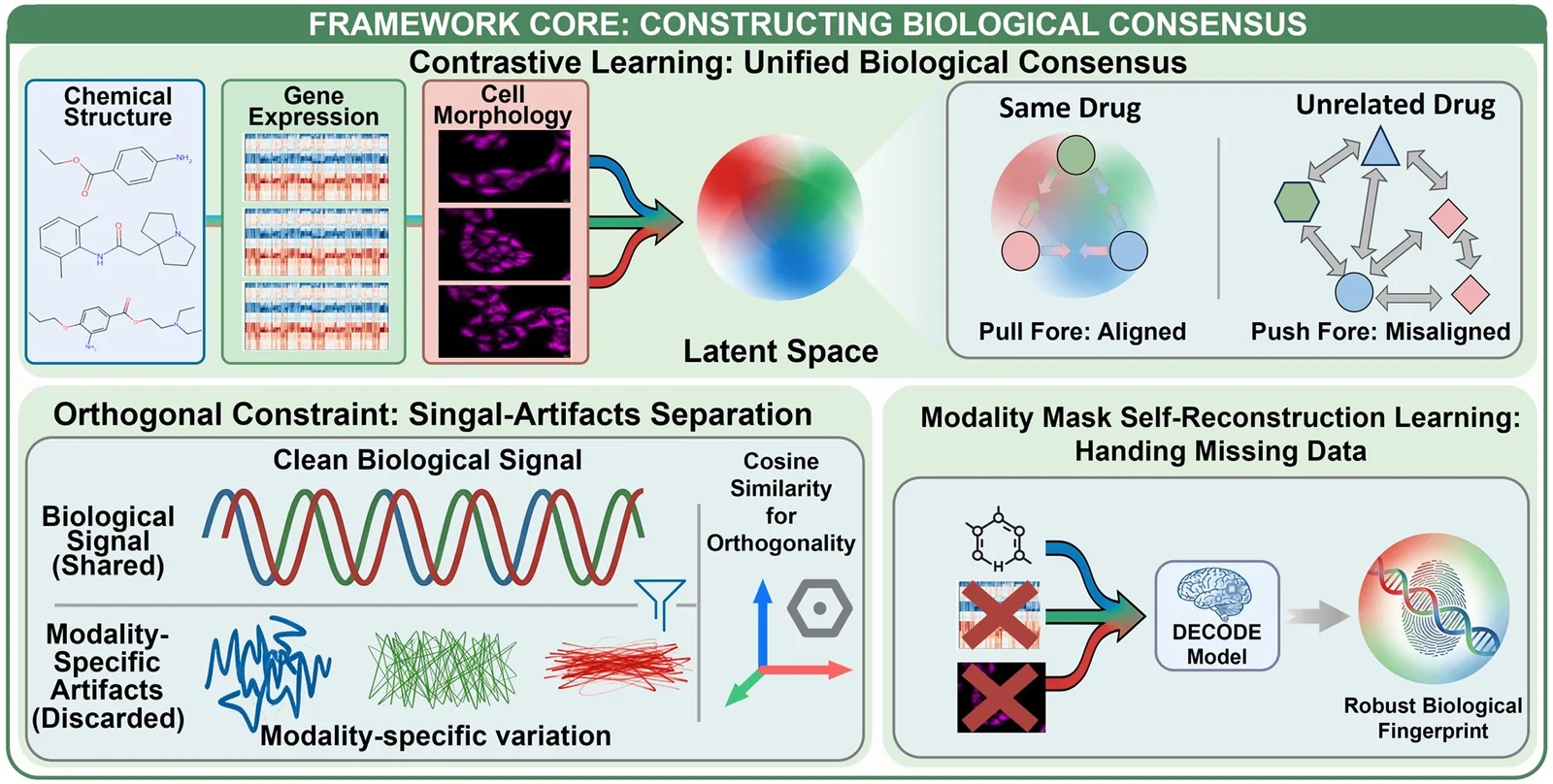
Overview of the DECODE framework. DECODE uses paired chemical structures, transcriptomic profiles, and morphological profiles during training to learn biologically informed molecular representations. Contrastive alignment encourages the shared components from different modalities to capture common response-related information, while orthogonal disentanglement separates shared signals from modality-specific artifacts and noise. Modality-mask self-reconstruction further improves robustness to missing biological profiles.

As shown in Fig. 1, DECODE aims to learn molecular representations enriched by biological profile supervision. Let $D={\left\{(x_{d}^{i},x_{g}^{i},x_{m}^{i},y^{i})\right\}}_{i=1}^{N}$ denote a multimodal dataset, where $x_{d}$ is the chemical structure, and $x_{g}\in R^{d_{g}}$ and $x_{m}\in R^{d_{m}}$ are transcriptomic and morphological profiles. Following the learning-with-privileged-information setting (Vapnik and Vashist 2009), $x_{g}$ and $x_{m}$ are used to supervise representation learning during training, while inference can be performed from chemical inputs without requiring newly measured biological profiles. The goal is to learn a representation $z=f_{\theta }(x_{d})$ that retains response-related biological information distilled from transcriptomic and morphological views.

### 2.2 Chemical representation encoding with optional dose context

The molecular structure $x_{d}$ serves as the anchor for drug identity. Given a molecular encoder $E_{\text{mol}}$, DECODE first maps $x_{d}$ to a molecular embedding:


(1) $$h_{d}=E_{\text{mol}}(x_{d}),\;h_{d}\in R^{h}.$$

We use MolFormer (Ross *et al.* 2022) as the default backbone and further evaluate VideoMol (Xiang *et al.* 2024) as an alternative encoder.

When dose information is available, a dose context gate modulates the molecular embedding:


(2) $$g=\sigma (W_{g}c_{\text{dose}}+b_{g}),\;e_{d}={\text{Encoder}}_{\text{drug}}(h_{d}⊙g),$$

Where $c_{\text{dose}}=E_{\text{dose}}(x_{\text{dose}})$. For downstream tasks without dose annotations, $c_{\text{dose}}$ is implemented as a learnable context vector optimized during training and fixed during inference.

Transcriptomic and morphological profiles are encoded by modality-specific MLP encoders:


(3) $$e_{g}={\text{Encoder}}_{\text{gene}}(x_{g}),\;e_{m}={\text{Encoder}}_{\text{morph}}(x_{m}).$$

All modality encoders are implemented as multilayer perceptrons and project chemical, transcriptomic, and morphological inputs into comparable latent embeddings for subsequent decomposition.

### 2.3 Geometric signal disentanglement and alignment

Biological profiles contain both drug-response signals and modality-specific variation, including batch effects, assay-specific artifacts, and measurement noise. DECODE therefore decomposes each modality embedding $e_{k}$ (k∈{d,g,m}) into a shared component and a modality-specific component:


(4) $$s_{k}=G_{s}(e_{k}),\;u_{k}=G_{u,k}(e_{k}).$$

The shared component $s_{k}$ is designed to capture response-related information that is consistent across chemical, transcriptomic, and morphological views and can be internalized into the chemical representation. The modality-specific component $u_{k}$ captures private variation of each modality, including technical artifacts/noise and potentially useful modality-specific biological information.

To separate the shared consensus space from modality-specific factors, we impose an orthogonality constraint:


(5) $$L_{\mathrm{ortho}}=\sum_{k}|\text{sim}(s_{k},u_{k})|+\sum_{i\neq j}|\text{sim}(u_{i},u_{j})|,$$

Where sim(·,·) denotes cosine similarity. The first term reduces redundancy between shared and modality-specific components within each modality, while the second term prevents modality-specific components from collapsing into the same subspace.

To transfer biological response information into the molecular representation, DECODE aligns the shared chemical component with both shared biological components, namely the transcriptomic component $s_{g}$ and the morphological component $s_{m}$, using an InfoNCE objective (Oord *et al.* 2018)


(6) $$L_{\mathrm{contrast}}=\frac{1}{2}\left(L_{d,g}+L_{d,m}\right),$$

(7) $$L_{d,k}=-\text{log}\;\frac{\;\text{exp}(\text{sim}(s_{d},s_{k})/\tau )}{\sum_{j=1}^{B}\;\text{exp}\;(\text{sim}(s_{d},s_{k}^{j})/\tau )},\;k\in \{g,m\},$$

Where τ is the temperature parameter and *B* is the batch size.

The final representation is obtained by aggregating the available shared and modality-specific components:


(8) $$z=\text{Concat}(\bar{s},\bar{u}),\;\bar{s}=\frac{1}{\left|V\right|}\sum_{k\in V}s_{k},\;\bar{u}=\frac{1}{\left|V\right|}\sum_{k\in V}u_{k},$$

Where *V* denotes the set of available modalities. The full pretraining objective is:


(9) $$L_{\mathrm{total}}=L_{\mathrm{recon}}+\lambda_{1}L_{\mathrm{ortho}}+\lambda_{2}L_{\mathrm{contrast}},$$

Where $L_{\mathrm{recon}}$ is an MMD-based reconstruction loss (Smola *et al.* 2006), and $\lambda_{1}$ and $\lambda_{2}$ control the orthogonality and contrastive terms.

### 2.4 Adaptive inference protocols

DECODE supports three inference protocols. The three downstream inference protocols are illustrated in Fig. S1, available as supplementary data at *Bioinformatics* online.

*Protocol I* uses chemical inputs to compute profile-distilled molecular representations for held-out functional retrieval, such as retrieving compounds with similar MoA. This protocol evaluates functional knowledge transfer from biological profiles to molecular representations and is not used as evidence of scaffold-disjoint chemical generalization.

*Protocol II* supports modality-flexible prediction by aggregating all available modalities through the same shared/specific decomposition, with modality masking used during training to improve robustness to missing profiles.

*Protocol III* performs profile-guided virtual screening. A frozen generator produces pseudo-biological features from the chemical input,


(10) $${\hat{x}}_{g},{\hat{x}}_{m}=G_{\text{frozen}}(x_{d}),$$

Which are deterministic functions of the molecular structure rather than independent experimental measurements. A task-specific integrator then combines these features with the molecular embedding:


(11) $$z_{\text{bio}}=F_{\text{trainable}}(h_{d},{\hat{x}}_{g},{\hat{x}}_{m}),\;\hat{y}=\text{MLP}(\text{Concat}(z_{\text{bio}},h_{d})).$$

This protocol reuses biological information learned during pretraining while adapting it to downstream screening tasks.

### 2.5 Data collection and evaluation protocols

DECODE was pretrained on LINCS and CDRP, which provide paired molecular structures, L1000 transcriptomic profiles, and Cell Painting morphological profiles (Bray *et al.* 2016, 2017, Subramanian *et al.* 2017, Natoli *et al.* 2021). These datasets were used to evaluate pretraining-stage representation quality through cross-modal reconstruction, CDRP held-out functional retrieval, and MoA prediction under different modality-availability settings.

Downstream evaluation was organized by task type. Binary virtual-screening tasks, including PRISM cancer activity prediction and four target-specific datasets (BACE1, COX-1, COX-2, and EP4), were used as the primary screening benchmarks. UMAP-clustering splits were used as the main chemically informed evaluation setting, while Bemis-Murcko scaffold splits were reported as complementary analyses (Bemis and Murcko 1996, Guo *et al.* 2025). We report AUROC together with screening-oriented metrics, including average precision, BEDROC20, and EF1% (Truchon and Bayly 2007). Multi-class and multi-label mechanism-annotation tasks, including PRISM-MOA classification and MCELC pathway prediction, were evaluated using UMAP clustering splits after class-support filtering and reported as secondary analyses.

External known-active lists were used only as supporting recovery analyses because they contain positives without naturally matched negatives. We standardized compounds at the parent-molecule level, removed salts and duplicate parents, and reported cleaned recovery results in the Supplementary Materials, available as supplementary data at *Bioinformatics* online (Fourches *et al.* 2010). Dataset details and implementation settings are provided in Supplementary Sections S1 and S2, available as supplementary data at *Bioinformatics* online. Baselines include MolFormer, VideoMol, MultiDCP, MIGA, capacity-matched large-head variants, and DECODE variants using MolFormer or VideoMol backbones (Ross *et al.* 2022, Wu *et al.* 2022, Xiang *et al.* 2024, Zheng *et al.* 2024).

## 3 Results

### 3.1 Latent-space analysis and cross-modal reconstruction

We first evaluated whether DECODE learns the intended shared and modality-specific representations. This analysis serves as a diagnostic of representation learning, rather than evidence that reconstructed profiles can replace measured transcriptomic or morphological profiles.

**Figure 2:**
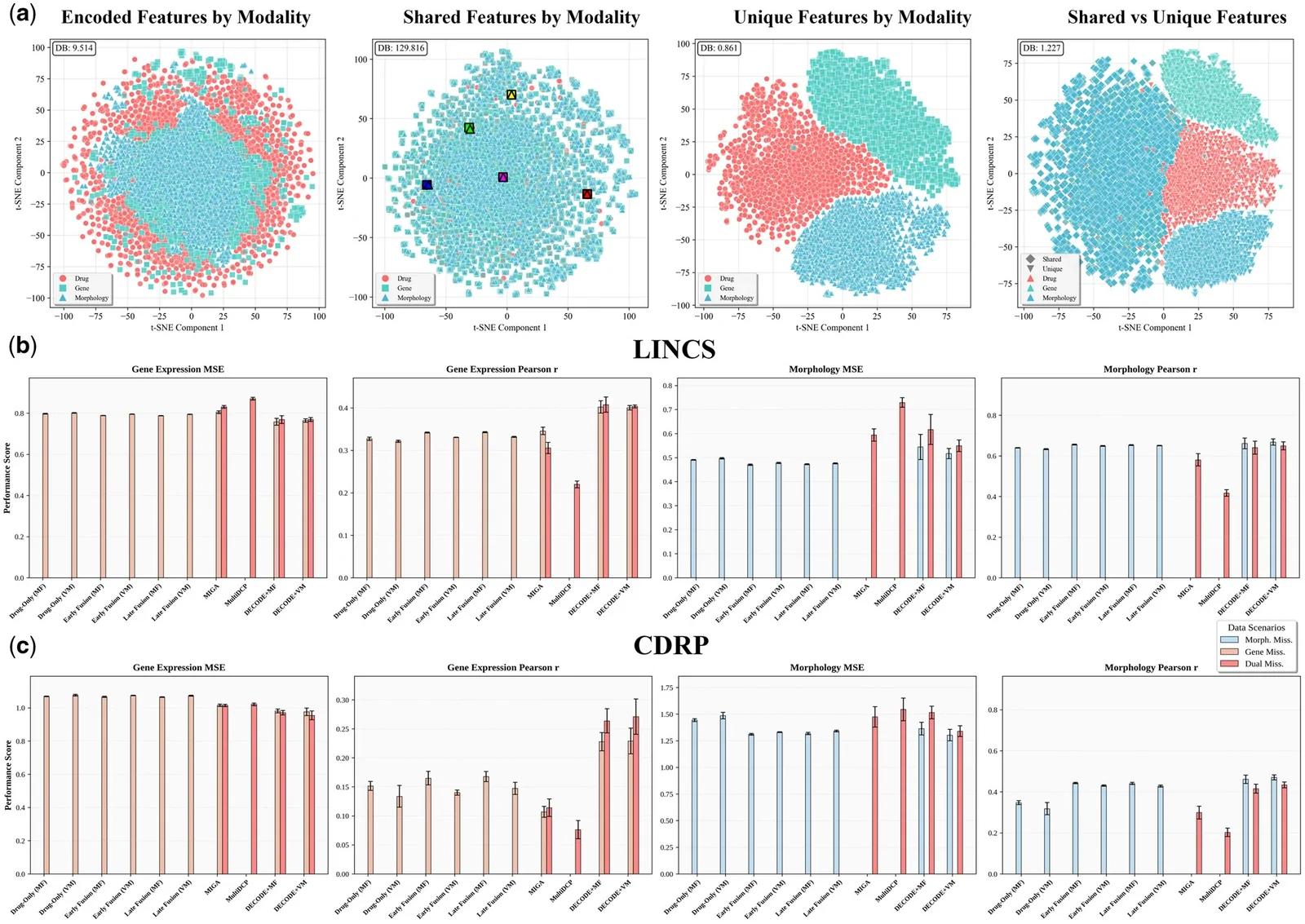
Representation-level analysis and cross-modal reconstruction on LINCS and CDRP. (a) t-SNE visualization of encoded features, shared features, modality-specific features, and shared-versus-specific features. The shared space shows stronger cross-modal mixing, whereas the modality-specific space preserves modality-dependent variation. (b, c) Reconstruction performance on LINCS and CDRP under different missing-modality scenarios. MSE and Pearson correlation are reported for transcriptomic and morphological reconstruction. Lower MSE and higher Pearson correlation indicate better reconstruction.

As shown in Fig. 2(a), the encoded features still show clear modality-dependent patterns. After disentanglement, the shared features across chemical, transcriptomic, and morphological views become more overlapping, indicating that DECODE aligns response-related information across modalities. In contrast, the unique features are more separated by modality, suggesting that modality-specific variation is captured outside the shared space. These results support the design of separating shared response signals from modality-dependent factors rather than directly mixing all modality information into a single representation.

We also assessed whether the learned representations preserve profile-related information using cross-modal reconstruction. On both LINCS and CDRP, DECODE achieves competitive reconstruction performance. This holds true compared with chemical-only, fusion-based, and multimodal baselines under different missing-modality settings (Fig. 2b and c). In particular, DECODE generally improves Pearson correlation for reconstructing missing gene-expression or morphological profiles. This suggests the learned representation retains biologically relevant profile information.

### 3.2 Held-out functional retrieval (Protocol I)

We next evaluated whether DECODE transfers functional information from biological profiles into chemical representations. For each query compound, candidates were ranked by representation similarity, and compounds with the same MoA label were treated as positives. This experiment is intended to evaluate functional knowledge transfer during pretraining, rather than to serve as the main evidence for chemical generalization.

**Figure 3:**
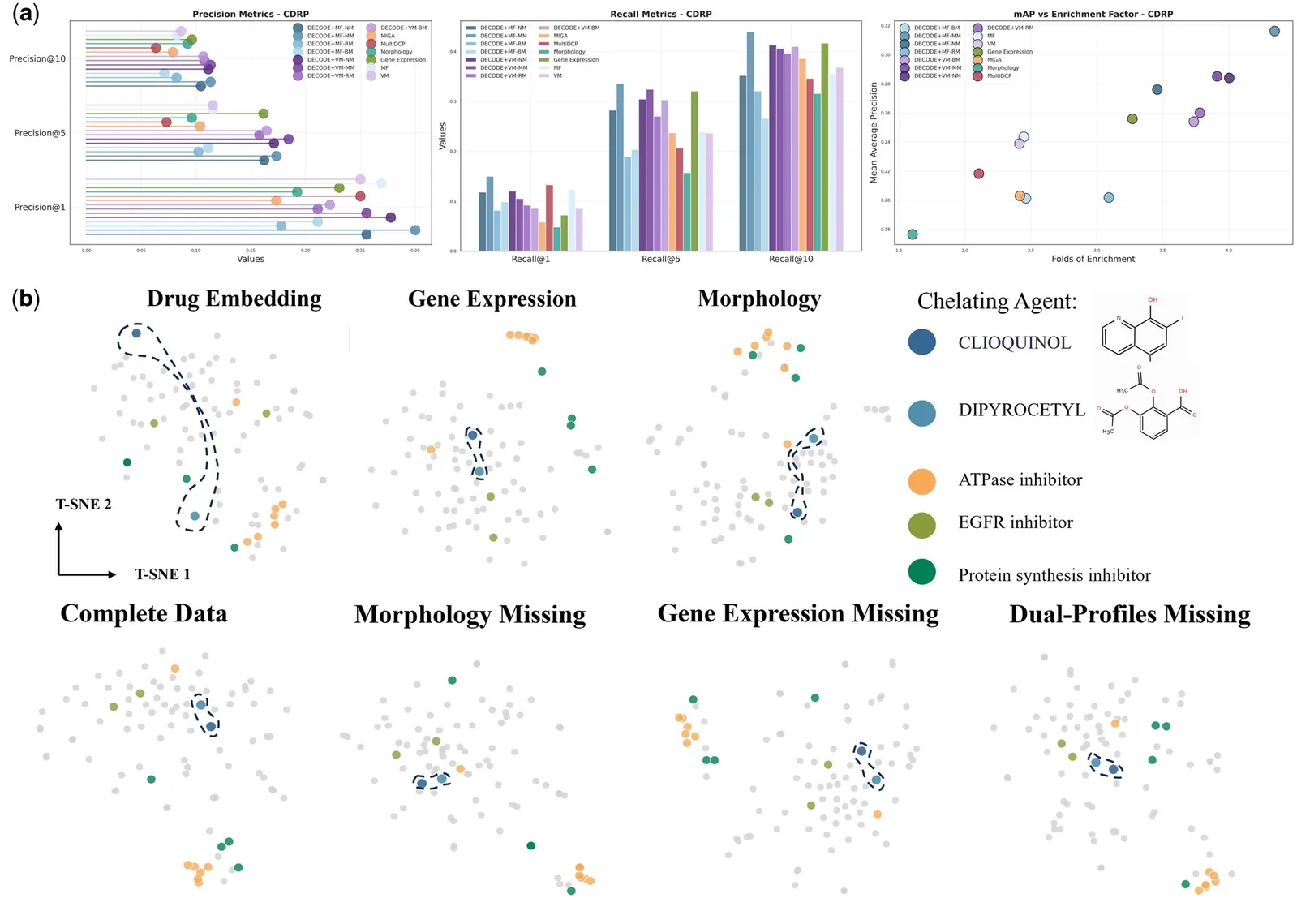
Held-out functional retrieval and functional alignment in Protocol I. (a) Retrieval performance on CDRP, measured by Precision@K, Recall@K, mean average precision, and enrichment factor. (b) t-SNE visualization of drug embeddings, measured biological profiles, and DECODE representations under different modality-availability settings. The highlighted chelating agents, CLIOQUINOL and DIPYROCETYL, are separated in the original drug embedding space but become closer in biological-profile spaces and DECODE representations. “Dual-Profiles Missing” denotes chemical-input inference without measured transcriptomic or morphological profiles.

As shown in Fig. 3a, DECODE achieves favorable retrieval performance on CDRP across precision, recall, mean average precision, and enrichment factor. Notably, the dual-profiles-missing setting remains competitive, although no measured transcriptomic or morphological profiles are provided at inference. This suggests that training-time biological profile supervision improves the chemical representation for function-oriented retrieval.

Figure 3b further illustrates this effect. The chelating agents CLIOQUINOL and DIPYROCETYL are distant in the original drug embedding space, but become closer in measured biological-profile spaces and in DECODE representations, including the dual-profiles-missing setting. This indicates that DECODE can capture functional similarity that is not fully reflected by structural embeddings alone. At the same time, compounds from heterogeneous MoA classes remain distributed across the latent space, suggesting that the model does not simply collapse all compounds with the same label into compact clusters.

We further examined stricter chemical partitioning for LINCS/CDRP retrieval in Supplementary Section S1.2, available as supplementary data at *Bioinformatics* online. This analysis shows that scaffold-based retrieval on these pretraining datasets is strongly affected by the functional separability of raw biological profiles and the available MoA label support. Therefore, we report Protocol I as a held-out functional retrieval diagnostic, while chemically stricter generalization is evaluated in the downstream virtual-screening benchmarks using scaffold and UMAP-clustering splits.

### 3.3 Modality-flexible MoA prediction (Protocol II)

We next evaluated supervised mechanism-of-action (MoA) prediction on the LINCS and CDRP datasets under different modality-availability settings. This experiment tests whether DECODE remains reliable when transcriptomic profiles or morphological profiles are partially missing, or when only the chemical input is available at inference.

**Figure 4:**
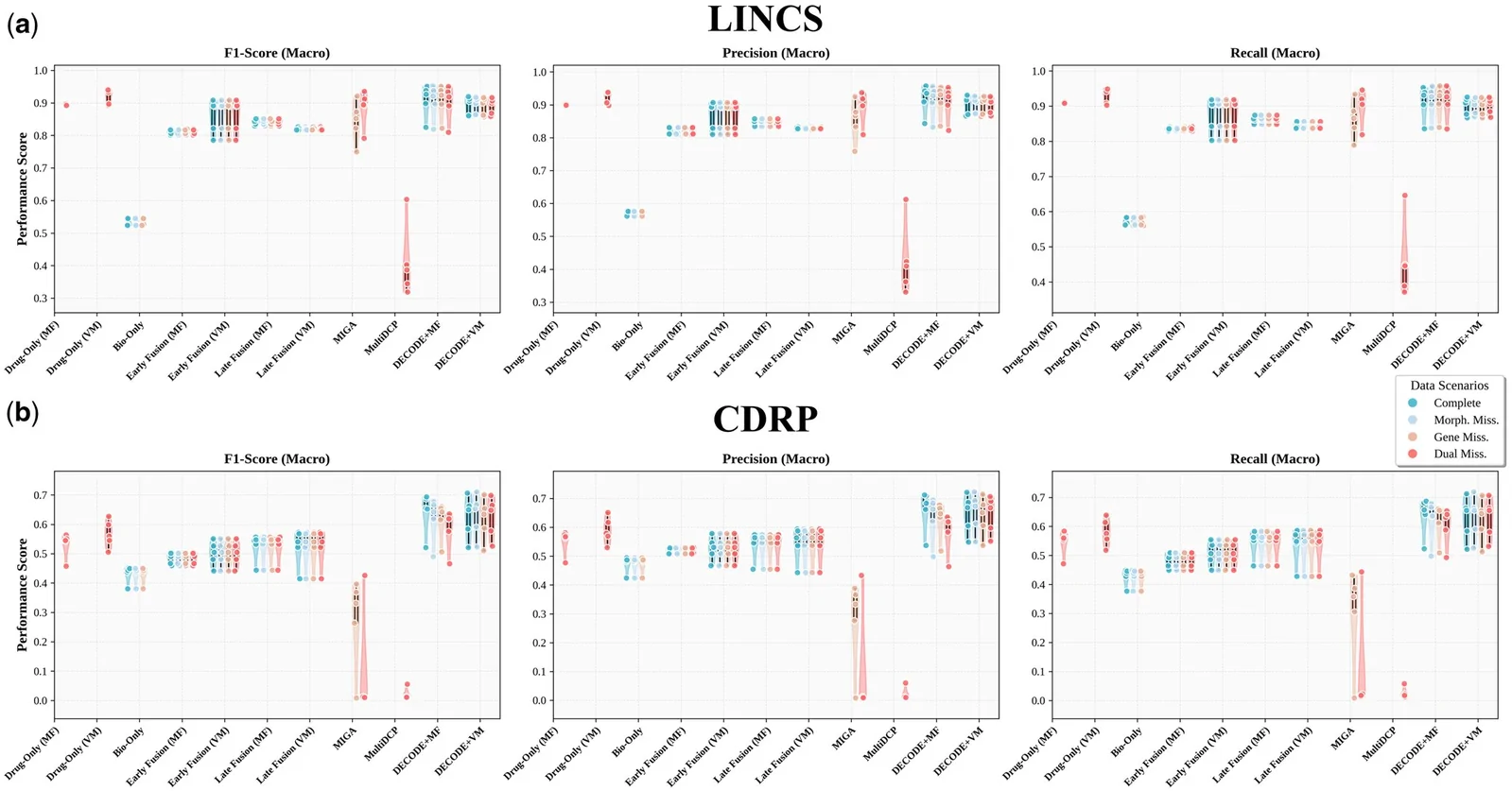
Modality-flexible MoA prediction on LINCS and CDRP. Macro F1-score, macro precision, and macro recall are reported under four modality-availability settings: complete data, morphology missing, gene-expression missing, and dual-profile missing. (a) Results on LINCS. (b) Results on CDRP. DECODE maintains stable performance across modality-availability settings, suggesting that the learned consensus representation can support both multimodal inference and biological-profile-free chemical inference.

As shown in Fig. 4, DECODE achieves favorable performance in macro F1-score, macro precision, and macro recall under various settings: complete-data, morphology-missing, gene-expression-missing, and dual-profile-missing. The small performance variation across these settings suggests that DECODE can function beyond conventional multimodal fusion. Traditional fusion typically assumes a fixed set of modalities. Instead, DECODE’s shared/specific decomposition lets the model use available biological profiles when present. When profiles are missing, it relies on the biologically informed chemical representation.

Compared with biological-only and fusion-based baselines, DECODE shows more stable behavior across changes in available modalities. This suggests that directly combining raw transcriptomic and morphological profiles can introduce modality-dependent variation, whereas DECODE extracts response-related signals that are shared across modalities and reduces the influence of modality-specific factors.

Together, these results support the role of geometric disentanglement in learning a modality-flexible representation for MoA prediction. Feature distribution analysis after supervised fine-tuning further shows that the learned consensus space remains stable after MoA optimization (Supplementary Section S5, available as supplementary data at *Bioinformatics* online).

### 3.4 Virtual screening under chemically informed splits (Protocol III)

We next evaluated whether DECODE improves downstream virtual screening. In Protocol III, pseudo-biological features are created from chemical inputs and then combined with molecular embedding for prediction. These features are deterministic functions of molecular structure. They serve to reuse biological information learned during pretraining.

**Figure 5:**
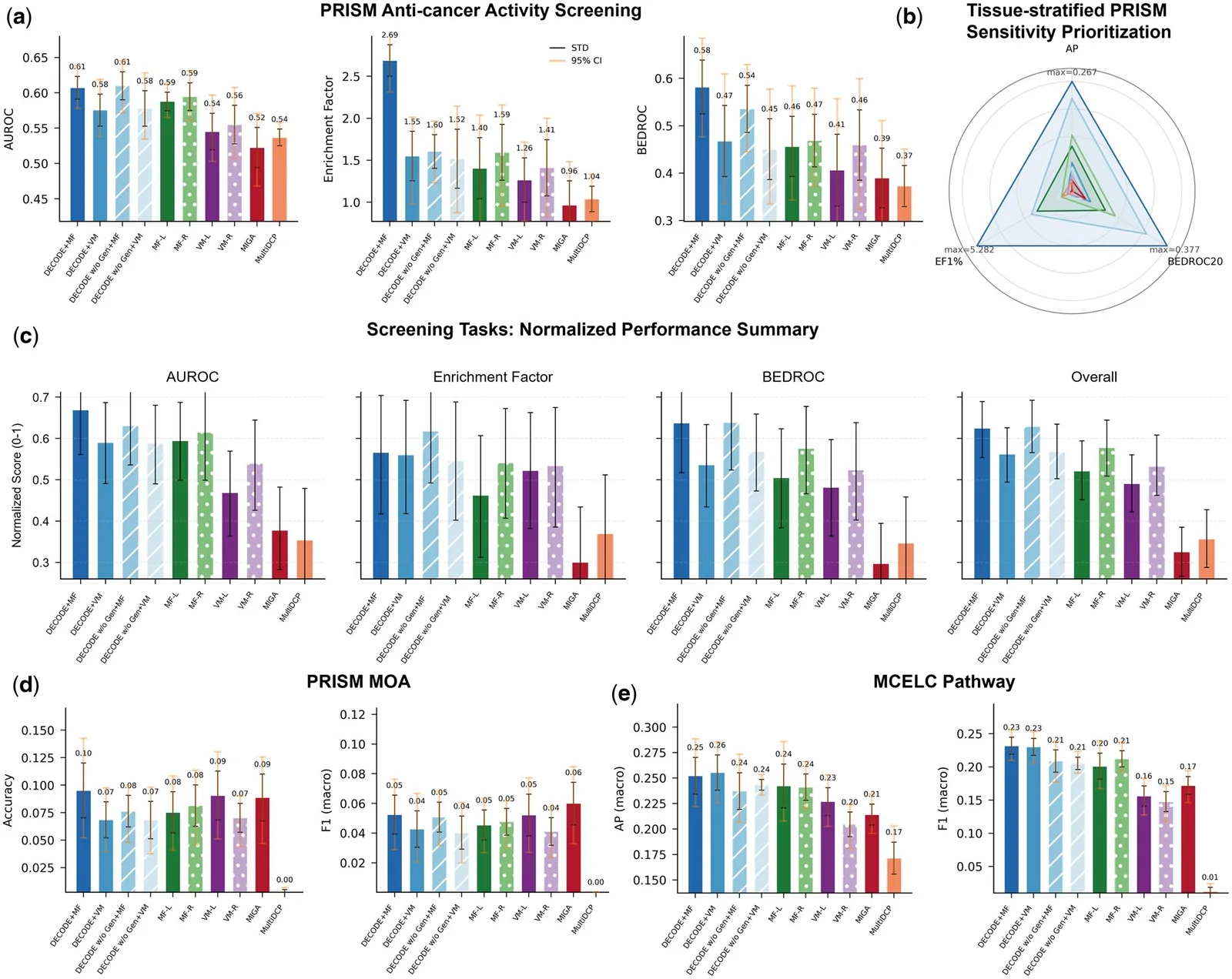
Downstream virtual-screening and mechanism-annotation evaluation under chemically informed settings. (a) PRISM anti-cancer activity screening evaluated by AUROC, EF1%, and BEDROC20. (b) Tissue-stratified PRISM sensitivity prioritization, where AP, BEDROC20, and EF1% are reported at the tissue level. (c) Normalized performance summary across binary screening tasks, including PRISM anti-cancer activity and target-specific ligand-screening benchmarks. Scores are normalized to [0,1] within each task and metric before aggregation. (d) PRISM-MOA classification under UMAP-clustering split, reported by accuracy and macro F1-score. (e) MCELC pathway prediction under UMAP-clustering split, reported by macro AP and macro F1-score. All enrichment factors are EF1%, and all BEDROC values are BEDROC20. MF and VM denote MolFormer and VideoMol backbones, respectively.

Binary screening tasks were used as the primary evaluation setting, including PRISM anti-cancer activity and target-specific ligand-screening benchmarks. Because virtual screening emphasizes early recognition, we report AUROC, EF1%, and BEDROC20 (Truchon and Bayly 2007). On PRISM anti-cancer activity screening, DECODE improves early-recognition performance over the corresponding molecular backbone and other molecular or multimodal baselines (Fig. 5a). Across binary screening tasks, the normalized summary also shows strong overall performance for DECODE variants, especially on EF1% and BEDROC20 (Fig. 5c). These comparisons include capacity-matched large-head variants, suggesting that the gain is not explained only by predictor capacity.

We further stratified PRISM by tumor tissue to evaluate compound ranking across diverse cancer types (Corsello *et al.* 2020). This approach does not predict tissue- or cell-line-specific responses, as it uses no such descriptors. Instead, DECODE relies on paired gene expression and cell structure profiles from pretraining, imposing drug-induced changes directly on molecular representations. Although this cannot capture all tissue-specific PRISM responses, it steers chemical representations toward mechanisms and observable effects, beyond just chemical similarity. Figure 5b shows that DECODE increases AP, BEDROC20, and EF1% within tissue groups. These results support tissue-stratified evaluation of compound ranking.

We also report PRISM-MOA classification and MCELC pathway prediction as secondary analyses under UMAP-clustering splits. PRISM-MOA is difficult for all models, with low macro F1-scores under clustered partitioning (Fig. 5d). In contrast, DECODE achieves a favorable AP and macro F1-score in MCELC pathway prediction (Fig. 5e). Full per-task metrics and cleaned external recovery results are provided in Supplementary Materials, available as supplementary data at *Bioinformatics* online.

### 3.5 Ablation and sensitivity analysis

We performed ablation and sensitivity analyses to examine the contributions of biological-profile supervision, geometric disentanglement, auxiliary reconstruction, and dose assumptions. Component-level ablations on LINCS and CDRP are summarized in Supplementary Section S7.1, available as supplementary data at *Bioinformatics* online. Removing modal alignment, contrastive consensus, or orthogonal disentanglement generally weakens the primary MoA prediction and retrieval metrics. This is especially true when biological profiles are partially or fully unavailable. These results support the use of paired transcriptomic and morphological profiles for training-time supervision. They also suggest that explicit shared/specific decomposition is more effective than simple fusion or joint reconstruction.

We further compared DECODE with a drug-only auxiliary-reconstruction initialization on cancer activity screening (Supplementary Section S7.2, available as supplementary data at *Bioinformatics* online). This control uses the same molecular input and downstream settings as DECODE but excludes paired biological-profile alignment and geometric decomposition, and performs worse than DECODE on ROC-AUC, average precision, and EF1%. We also evaluated fixed-dose, marginalized-dose, learnable-dose, random-dose, and no-dose settings (Supplementary Section S7.3, available as supplementary data at *Bioinformatics* online). The performance changes remain small across cancer activity prediction and held-out retrieval, indicating that the reported gains are not driven by auxiliary reconstruction alone or by a specific inference-time dose choice.

## 4 Conclusion

This work presents DECODE, a framework for learning biologically informed molecular representations from paired chemical, transcriptomic, and morphological data. By using biological profiles as training-time privileged information, DECODE transfers cellular response-related signals into chemical representations while preserving biological-profile-free inference. Through contrastive alignment, geometric disentanglement, and modality-mask reconstruction, DECODE improves functional retrieval, modality-flexible MoA prediction, and downstream virtual screening. Under chemically informed scaffold and UMAP-clustering splits, DECODE achieves favorable early-recognition performance, suggesting that biological-profile supervision helps molecular representations capture response-related drug properties beyond structural similarity alone.

## 5 Limitations and future work

DECODE currently generates pseudo-biological features from chemical inputs, which support downstream prediction but do not replace measured transcriptomic or morphological profiles. The current framework also focuses on drug-level representations and does not explicitly model cell-line, tissue, or disease-context information. Future work will incorporate context-aware conditioning to better capture heterogeneous drug responses. Another promising direction is to integrate biological foundation models as denoising and semantic feature extractors, reducing batch effects and assay-specific noise in raw profiles while providing richer supervision for phenotype-aware virtual screening.

## Supplementary Material

btag517_Supplementary_Data

## Data Availability

The data and source code of DECODE are available at https://github.com/lian-xiao/DECODE.
