# Supplementary material for: Empowering chemical structures with biological insights for scalable phenotypic virtual screening
Source: Bioinformatics. 2026 Jul 25;42(8):btag517. doi: 10.1093/bioinformatics/btag517 (PMC13447017; doi:10.1093/bioinformatics/btag517)
Supplement: Supplementary file 1 [file btag517_supplementary_data.pdf]

Empowering Chemical Structures with Biological Insights for Scalable Phenotypic Virtual Screening

Xiaoqing Lian Pengsen Ma Tengfeng Ma Zhonghao Ren Xibao Cai Zhixiang Cheng He Wang Xiang Pan Bosheng Song Yangyang Chen Sisi Yuan* Chen Lin**

Xiaoqing Lian, Pengsen Ma, Tengfeng Ma, Zhonghao Ren, Xibao Cai, Zhixiang Cheng, Bosheng Song
State Key Laboratory of Chemo and Biosensing, College of Computer Science and Electronic Engineering, Hunan University, Changsha 410082, China
The Ministry of Education Key Laboratory of Fusion Computing of Supercomputing and Artificial Intelligence, Hunan University, Changsha 410082, China
He Wang, Xiang Pan
School of Artificial Intelligence and Computer Science, Jiangnan University, Wuxi 214122, China
Yangyang Chen
Department of Computer Science, University of Tsukuba, Tsukuba, Ibaraki, Japan
Email Address: chen.yangyang.xp@alumni.tsukuba.ac.jp
Sisi Yuan
School of Chinese Medicine, Hong Kong Baptist University, Kowloon Tong, Kowloon, Hong Kong SAR 999077, China
Email Address: sisiyuan@hkbu.edu.hk
Chen Lin
School of Informatics, National Institute for Data Science in Health and Medicine, Xiamen University, Xiamen 361000, China
Email Address: cheyenne.lin@foxmail.com

Contents

S1 Dataset Details **2**
S1.1 Pre-training Datasets . . . 2
S1.2 Effect of Chemical Partitioning on LINCS/CDRP Functional Retrieval . . . 2
S1.3 Downstream Datasets . . . 3
S1.4 Data partitioning and external-set curation. . . . 4

S2 Implementation Details **5**

S3 Downstream Inference Protocols **6**

S4 Visualization of Multi-modal Reconstruction Performance **8**

S5 Stability of the Consensus Space during Fine-tuning **9**

S6 Supplementary Evaluation for Downstream Virtual Screening **11**
S6.1 Target-specific ligand screening under UMAP-clustering splits . . . 11
S6.2 Cancer activity screening and external known-active recovery . . . 11
S6.3 Target-specific ligand screening under scaffold splits . . . 11

S7 Ablation and Sensitivity Analyses **15**
S7.1 Component-level ablation on LINCS and CDRP . . . 15
S7.2 Probing shared and modality-specific components . . . 16
S7.3 Auxiliary reconstruction control in cancer activity screening . . . 16
S7.4 Dose sensitivity analysis . . . 17

S1 Dataset Details

This study uses several public and internal datasets to comprehensively evaluate the performance, generalization, and practical applicability of the DECODE framework. This section provides a detailed description of these datasets.

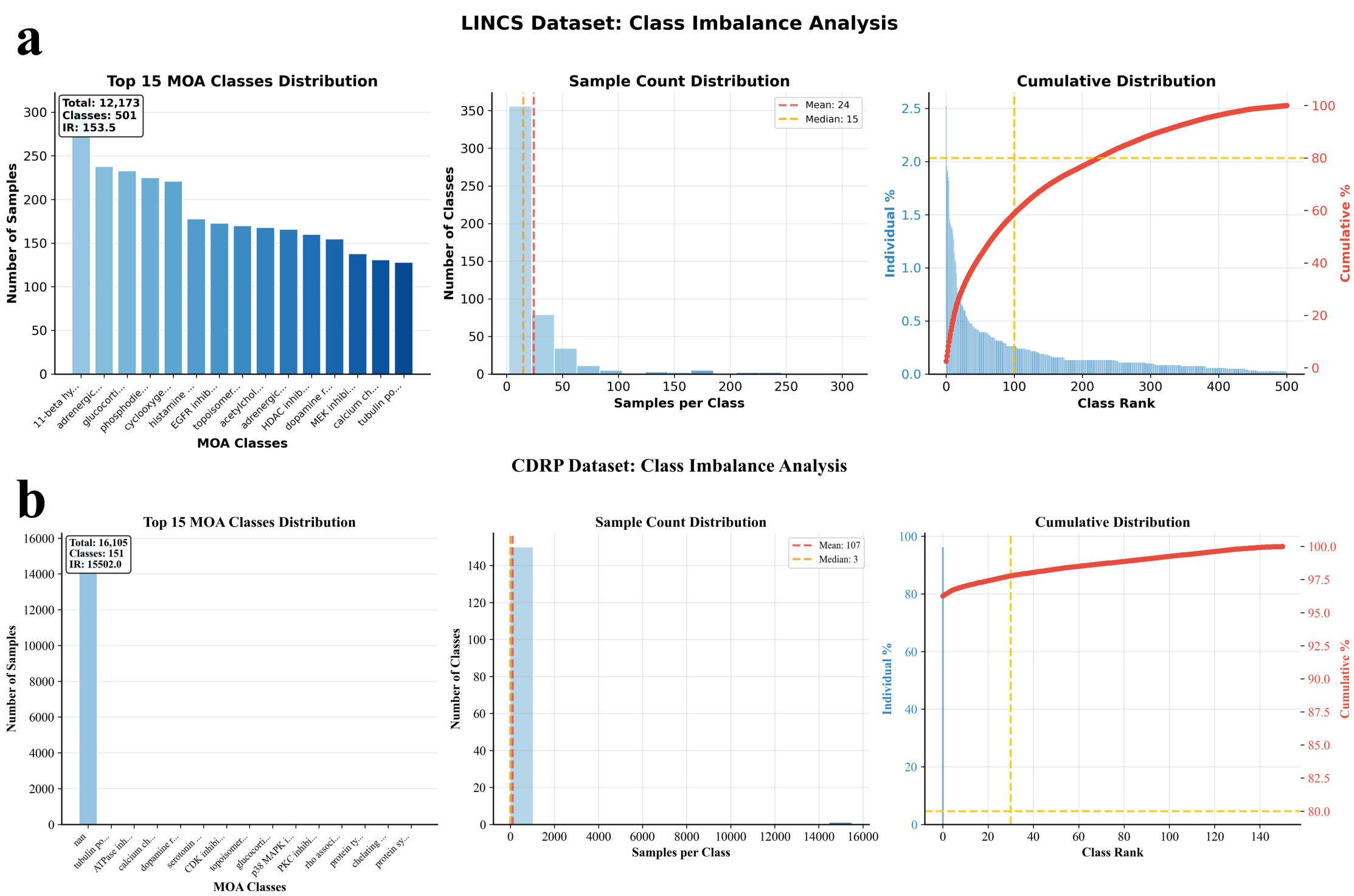

Figure S1: Analysis of the class imbalance in MOA categories for the LINCS and CDRP datasets. (a) The LINCS dataset exhibits a skewed distribution, with certain classes strongly overrepresented. (b) The CDRP dataset exhibits a highly imbalanced class distribution, reflecting real-world scenarios with many unknown MOAs.

S1.1 Pre-training Datasets

Two large-scale, multi-modal public datasets from the Broad Institute were primarily used for model pre-training: LINCS [1] and CDRP [2]. The distribution of Mechanism of Action (MOA) classes for these datasets is illustrated in Fig. S1. The LINCS dataset exhibits a long-tail distribution, with the top 100 most frequent MOA classes accounting for 80% of the total samples. This enables the model to learn from a rich, well-annotated set of biological effects. In contrast, the CDRP dataset presents a more challenging scenario that closely mimics real-world drug discovery, with over 95% of its samples having an unknown MOA. This data distribution simulates the early stages of drug screening, where the biological functions of numerous compounds have yet to be determined, thereby rigorously testing the model's unsupervised and representation-learning capabilities.

S1.2 Effect of Chemical Partitioning on LINCS/CDRP Functional Retrieval

We examined whether Protocol I retrieval can be directly evaluated under more stringent chemical partitioning conditions. On LINCS, scaffold splitting removes chemical overlap [3], but raw transcriptomic and morphological features show limited same-MoA retrieval and nearest-neighbor separability (Figure S2(a,b)). Therefore, scaffold retrieval on LINCS is affected not only by chemical extrapolation but also by the weak MoA separability of the measured biological profiles.
On CDRP, scaffold splitting substantially reduces usable retrieval support because known MoA labels are sparse. After query/gallery filtering, only a small number of test samples and MoA classes remain

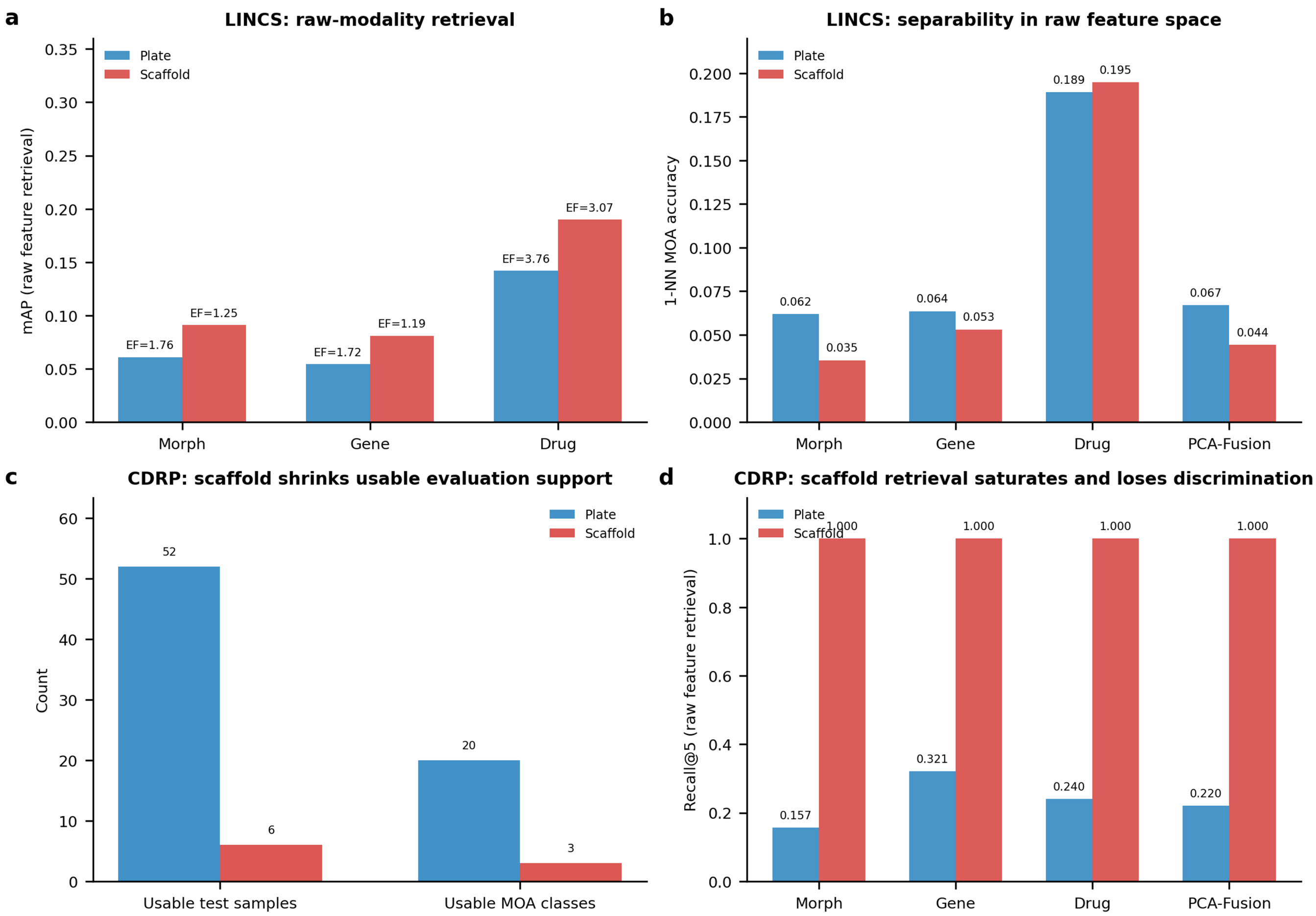

Figure S2: Effect of chemical partitioning on LINCS/CDRP functional retrieval. (a) Same-MoA retrieval using raw LINCS modality features under plate and scaffold splits. (b) One-nearest-neighbor MoA classification accuracy in the raw LINCS feature space. (c) Number of usable test samples and MoA classes for CDRP retrieval after query/gallery filtering. (d) Recall@5 of raw CDRP modality features under plate and scaffold splits. Scaffold partitioning substantially reduces usable CDRP retrieval support and leads to saturated Recall@5 values, making representation comparisons less discriminative.

(Figure S2(c)), and Recall@5 becomes saturated across raw modality features (Figure S2(d)). This makes scaffold retrieval less discriminative for comparing representation quality.
For these reasons, Protocol I is reported as a held-out functional retrieval diagnostic on the pretraining datasets. Scaffold-disjoint and clustering-based chemical generalization are evaluated in downstream binary virtual-screening tasks with explicit positive and negative labels [3; 4].

S1.3 Downstream Datasets

For the tasks of MOA prediction and anti-cancer activity screening in a novel cellular context, we employed the dataset from the PRISM drug repurposing screen, as introduced by Corsello et al. in Nature Cancer [5]. This dataset is a large-scale public resource designed to discover the anti-cancer potential of non-oncology drugs systematically. The screen profiled 4,518 compounds sourced from the Drug Repurposing Hub. This diverse library comprises 3,466 (77%) non-oncology drugs, 949 (21%) oncology-targeted agents, and 90 (2%) conventional chemotherapeutics; notably, 74% of these are approved or clinical-stage compounds. The compounds were screened against a panel of 578 adherent human cancer cell lines representing 24 distinct tumor types, of which 489 passed quality control for the secondary screening phase. The study used the PRISM molecular barcoding technology, in which cell viability is inferred from the abundance of barcoded mRNA. A two-stage screening process was implemented: (1) a primary screen at a single 2.5μM dose identified 1,448 active compounds, which were (2) subsequently advanced to a secondary screen featuring an 8-point dose-response curve (ranging from 610pM to 10μM)

to generate precise IC_{50} and AUC (Area Under Curve) values. The dataset includes the raw and processed activity values (AUC), known MOAs for 640 of the screened drugs, and comprehensive multi-omic characterizations of the cell lines. In our work, this dataset serves as a stringent test for generalization, used for both MOA classification and the binary classification of anti-cancer activity (active vs. inactive).

To evaluate the model's ability to predict therapeutic pathways in a disease-specific context, we used the MCE Anti-Lung Cancer Compound Library available on the MedChemExpress (MCE) website (`https://www.medchemexpress.com/`). The MedChemExpress offers a wide range of high-quality research chemicals and biochemicals, including novel life science reagents, reference compounds, and natural compounds for laboratory and scientific purposes [6; 7]. Lung cancer is the leading cause of cancer mortality, with non-small cell lung cancer (NSCLC) representing approximately 85% of all cases [8]. This curated library contains 2,449 compounds that possess documented or potential anti-lung cancer activity. The compounds are specifically selected to target key signaling pathways and driver oncogenes known to be critical in lung cancer, particularly NSCLC. These targets include, but are not limited to, EGFR and BRAF mutations, as well as ALK and ROS1 fusion proteins, all of which are established targets for targeted therapies. This dataset is employed in our study to predict compound activity against specific lung cancer therapeutic pathways.

Notably, to ensure training stability and result reliability, we filtered the classes for both the pathway and MOA prediction tasks, removing those with insufficient sample counts. The details of the two datasets used for anti-cancer drug screening are presented in Table S2.

For ligand-based virtual screening against specific protein targets, we used datasets curated and processed by the authors of the VideoMol study [9]. These datasets were initially sourced from the ChEMBL database [10]. The evaluation was conducted on four distinct and well-characterized therapeutic targets: Beta-secretase 1 (BACE1) with 6,860 molecules, Cyclooxygenase 1 (COX-1) with 3,423 molecules, Cyclooxygenase 2 (COX-2) with 4,988 molecules, and Prostaglandin E receptor 4 (EP4) with 350 molecules. The data curation process, as described in the original VideoMol publication, involved several key steps. First, only molecules with reported IC50 values were retained. Second, these IC50 values were converted to binary class labels (1 for active, 0 for inactive) using a specific threshold for each target. The details for these are provided in Table S3.

S1.4 Data partitioning and external-set curation.

We used independent partitioning protocols for the pretraining and downstream evaluation datasets. For LINCS and CDRP, we conducted model pretraining and pretraining-stage evaluation using independent plate-based splits within each dataset. For downstream virtual-screening benchmarks, we split each dataset independently using chemically informed partitions. UMAP-clustering splits served as the primary evaluation, and Bemis–Murcko scaffold splits were used as complementary analyses [11; 3; 4]. These downstream splits were constructed within each benchmark to separate training, validation, and test compounds according to the corresponding chemical partitioning rule.

External known-active drug sets were used as supporting recovery analyses rather than full virtual-screening benchmarks, because they contain curated active compounds without naturally matched negative samples. To reduce chemical redundancy, all compounds were standardized at the parent-molecule level, followed by salt and counterion removal, canonical SMILES generation, and InChIKey-based deduplication [12; 13; 14; 15]. The cleaned compound lists are provided in Tables S4-S8. These external sets were used to examine whether trained models assign high activity scores to known active compounds, while formal screening metrics such as AUROC, BEDROC20, and EF1% were computed only on binary benchmark datasets with explicit positive and negative labels [16].

S2 Implementation Details

This section summarizes the implementation details of DECODE and the compared baselines. All models were evaluated under the same dataset splits, validation protocols, and metrics described in the main text. Hyperparameters were selected using the validation set only, and the test set was used only for final evaluation.

DECODE architecture. The core components of DECODE are implemented with multilayer perceptrons (MLPs) using ReLU activation, batch normalization, and dropout. The transcriptomic and morphological encoders use hidden dimensions of [1024, 512]. The dose embedding has 64 dimensions and is used as an experimental-context embedding. It is not used to claim continuous interpolation between discretely sampled concentration anchors. The decoders for profile reconstruction use hidden dimensions of [512, 1024]. The fused representation is projected to a 256-dimensional latent vector, which is further decomposed into shared and modality-specific components using encoders with hidden dimensions of [256, 128]. Unless otherwise specified, the downstream classifier uses hidden dimensions of [512, 256].

Pretraining and fine-tuning. DECODE was pretrained separately on LINCS and CDRP using paired chemical, transcriptomic, and morphological profiles. The pretraining objective included reconstruction, contrastive alignment [17], and orthogonality losses, with weights set to 1.0, 1.0, and 0.5, respectively. No supervised classification loss was used during pretraining. We used AdamW with an initial learning rate of 5×10^{-4}, weight decay of 10^{-4}, batch size of 256, and a ReduceLROnPlateau scheduler with a reduction factor of 0.5 and patience of 5 epochs. Early stopping was applied when the validation loss did not improve for 15 consecutive epochs.
For supervised MoA prediction and downstream screening tasks, the pretrained weights were used for initialization and the model was optimized with the task-specific classification loss. Pretraining losses were disabled during this supervised fine-tuning stage. For virtual-screening tasks, the classifier head was extended to [512, 256, 128], and the learning rate was selected from the validation set. Unless dose information was explicitly provided by a dataset, dose was treated as a predefined experimental context variable rather than a test-time biological input. In the default downstream setting, the learnable dose context is a task-adaptive token optimized on the training split and fixed at test time; it is not selected using test labels and is not intended to extrapolate a continuous dose-response curve. The influence of this choice was examined separately through fixed-dose, randomized-dose, marginalized-dose, learnable-dose, and no-dose sensitivity analyses.

Backbone variants. DECODE was evaluated with two molecular backbones. DECODE(MF) uses MolFormer embeddings [18] as molecular input, while DECODE(VM) uses VideoMol embeddings [9]. For DECODE(VM), the input dimensionality and hidden dimensions were adjusted to match the VideoMol representation size, while keeping the same overall DECODE design, including biological-profile supervision, shared/specific decomposition, and modality-masking reconstruction.

Baseline implementation. We compared DECODE with chemical-only, biological-only, early-fusion, late-fusion, capacity-matched large-head, and recent molecular or multimodal baselines. MolFormer [18] and VideoMol [9] baselines were trained under the same downstream split and model-selection protocol as DECODE. Their large-head variants were included to control for the possibility that performance gains come from a more expressive classifier rather than biological-profile supervision.
For MIGA [19] and MultiDCP [20], we followed the released implementations and original training designs as closely as possible. Necessary adaptations were limited to dataset formatting, task-specific output heads, and modality-availability settings required by our evaluation protocol. We did not introduce DECODE-specific components into these baselines. When an original method did not naturally support a particular missing-modality scenario, we reported the closest applicable setting and used the same validation-based model-selection rule.

Evaluation protocol. For LINCS and CDRP pretraining-stage evaluation, models were assessed on reconstruction, held-out functional retrieval, and MoA prediction under different modality-availability settings. For downstream virtual screening, all methods were evaluated on the same chemically informed splits, including Bemis–Murcko scaffold splits and UMAP-clustering splits where applicable [3; 11; 4]. Binary screening tasks were evaluated using AUROC, average precision, BEDROC20, and EF1% [16]. Multi-class and multi-label mechanism-annotation tasks were evaluated using macro-averaged classification metrics. External known-active lists were standardized and deduplicated at the parent-molecule level [12; 13; 14; 15] and were used only as recovery analyses, not as full binary benchmarks.
A summary of the compared baseline families, their input modalities, and adaptation rules is provided in Table S9.

S3 Downstream Inference Protocols

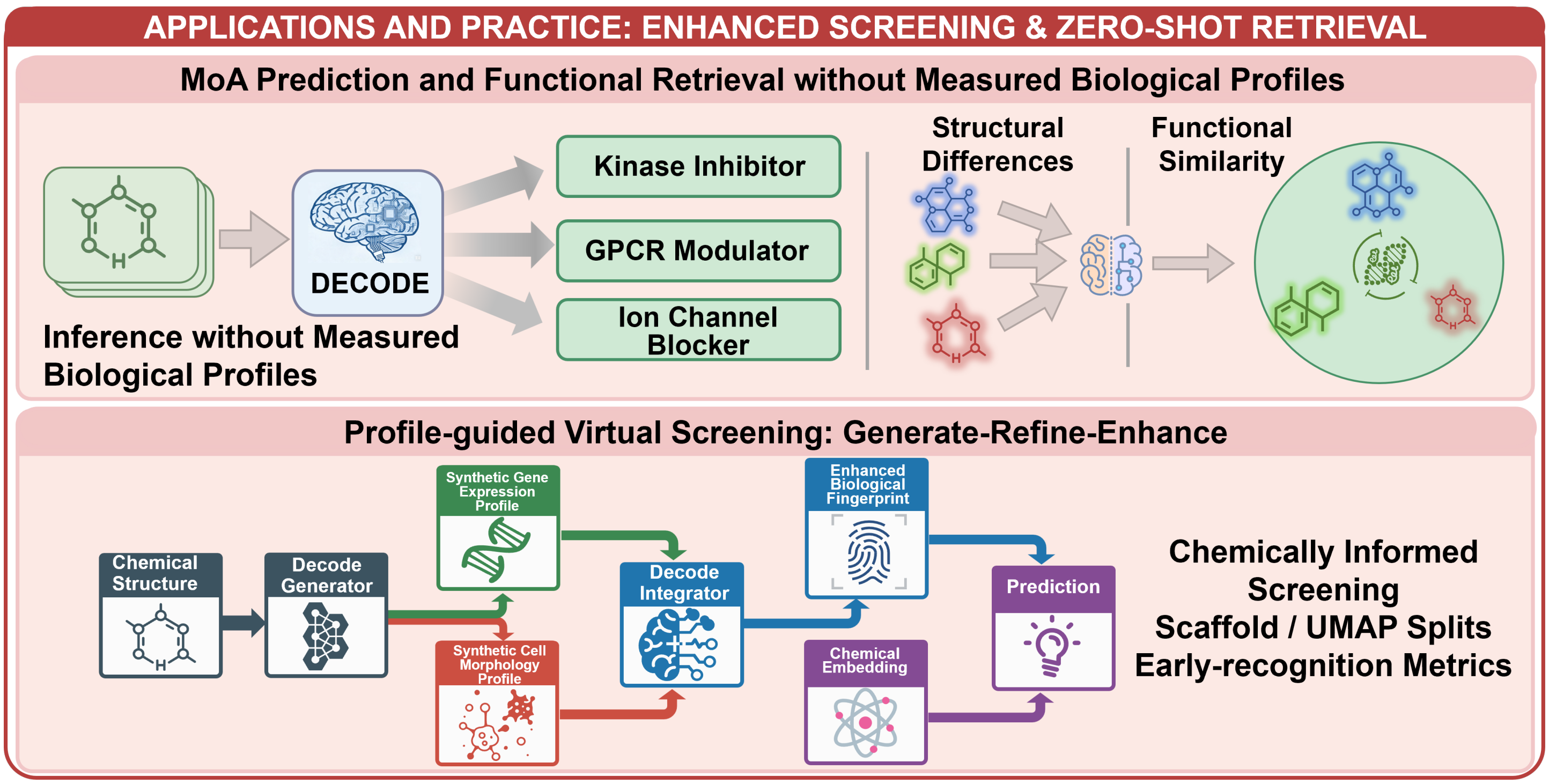

Figure S3: Conceptual overview of downstream inference protocols supported by DECODE. The upper panel illustrates chemical-input functional retrieval and MoA prediction, where DECODE uses molecular structures to retrieve functionally related compounds or predict MoA labels without requiring measured transcriptomic or morphological profiles at inference. The lower panel illustrates profile-guided virtual screening, where pseudo-gene-expression and pseudo-morphology features generated from chemical inputs are refined with molecular embeddings for task-specific activity prediction. The right part highlights the intended use of chemically informed screening evaluation, including scaffold and UMAP-clustering splits with early-recognition metrics such as AP, BEDROC20, and EF1% [3; 4; 16].

Supplementary Figure S3 provides a conceptual overview of the three downstream inference protocols supported by DECODE. Protocol I uses chemical inputs for held-out functional retrieval. Protocol II supports modality-flexible MoA prediction when different subsets of chemical, transcriptomic, or morphological information are available. Protocol III performs profile-guided virtual screening by generating pseudo-biological features from chemical inputs and integrating them with molecular embeddings for task-specific prediction. This figure summarizes the application settings of DECODE and is not used as quantitative evidence for model performance.

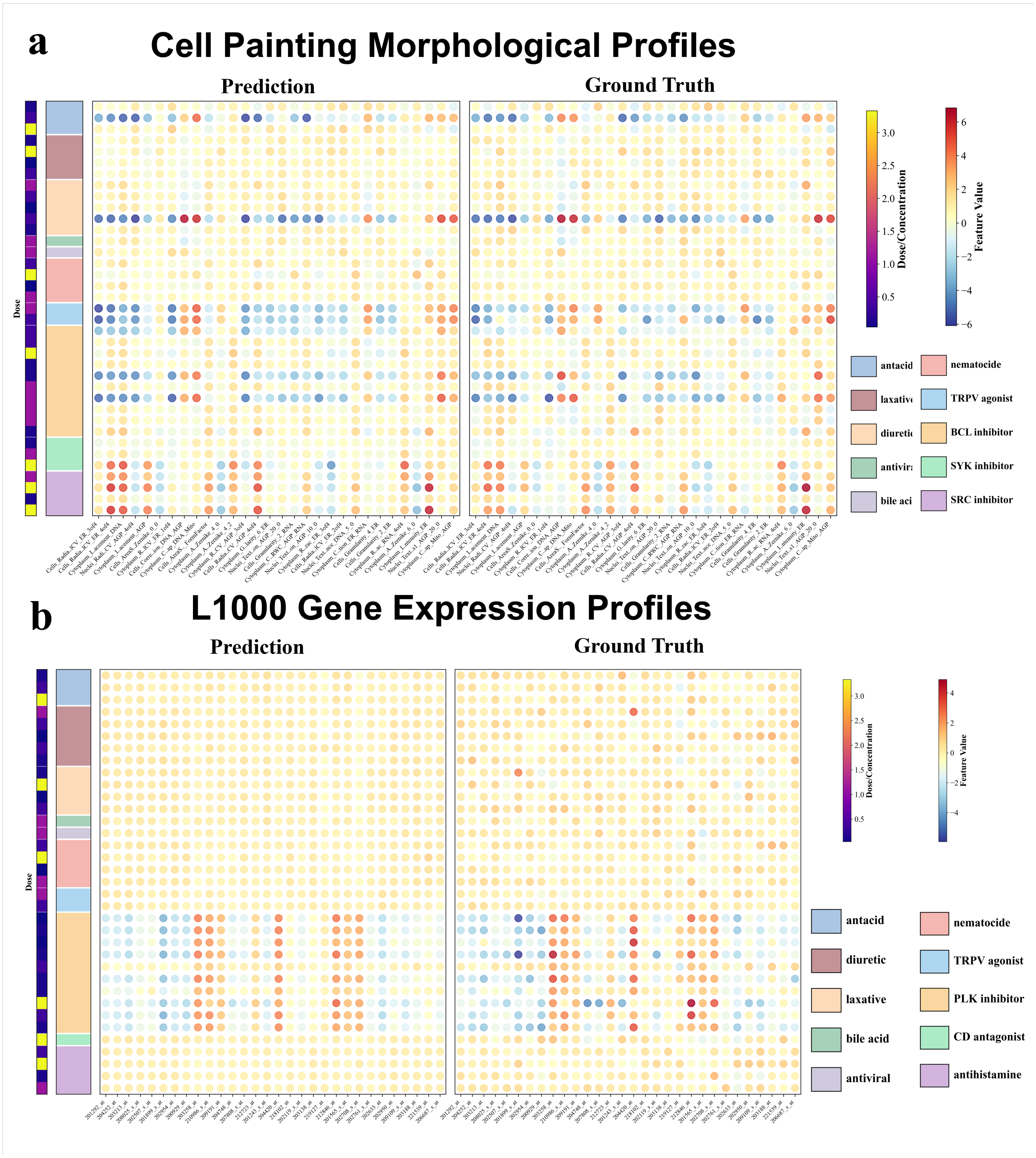

Figure S4: (a) Heatmap showing high correspondence between ground-truth gene expression profiles and those reconstructed by DECODE in a gene expression-missing scenario. The model accurately captures phenotypic signatures for different drug MOAs and dosages. (b) Violin plots showing that the statistical distribution of DECODE's predicted values aligns closely with the ground-truth distribution, both for the entire dataset and for specific MOA-defined subsets.

S4 Visualization of Multi-modal Reconstruction Performance

To qualitatively assess the generative capacity of DECODE, reconstructed phenotypic profiles were visualized alongside ground-truth measurements (Fig. S4). For morphological data, DECODE accurately captures the spatial distribution and intensity variations of cellular components across multiple channels, thereby reflecting mechanism of action (MoA)-specific phenotypic changes. In transcriptomic reconstruction, although gene expression data are inherently stochastic, the model effectively recovers the primary transcriptional response signatures. These findings indicate that DECODE's structural encoder internalizes the structure-to-phenotype mapping.

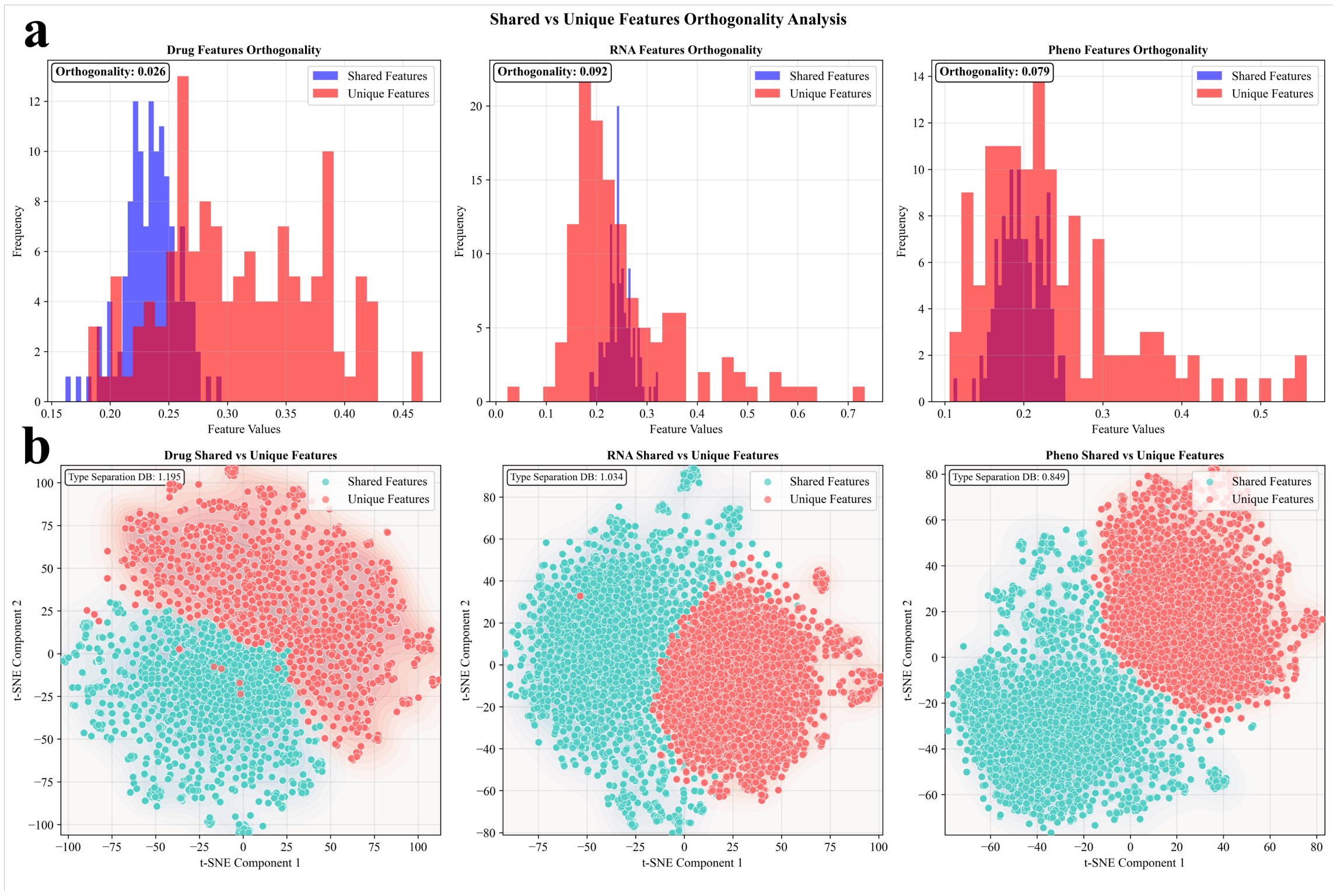

Figure S5: Modality alignment and feature stability of DECODE. (a) Frequency distribution of mean feature values across modalities. (b) Multi-modal latent space visualization via t-SNE.

S5 Stability of the Consensus Space during Fine-tuning

A feature distribution analysis was conducted to investigate the impact of task-specific fine-tuning on the learned consensus manifold. Supplementary Figure S5 shows that the alignment between structural and biological embeddings remains stable after model optimization for MoA classification. This observation suggests that the biological semantics distilled during pre-training are partly preserved during downstream fine-tuning.

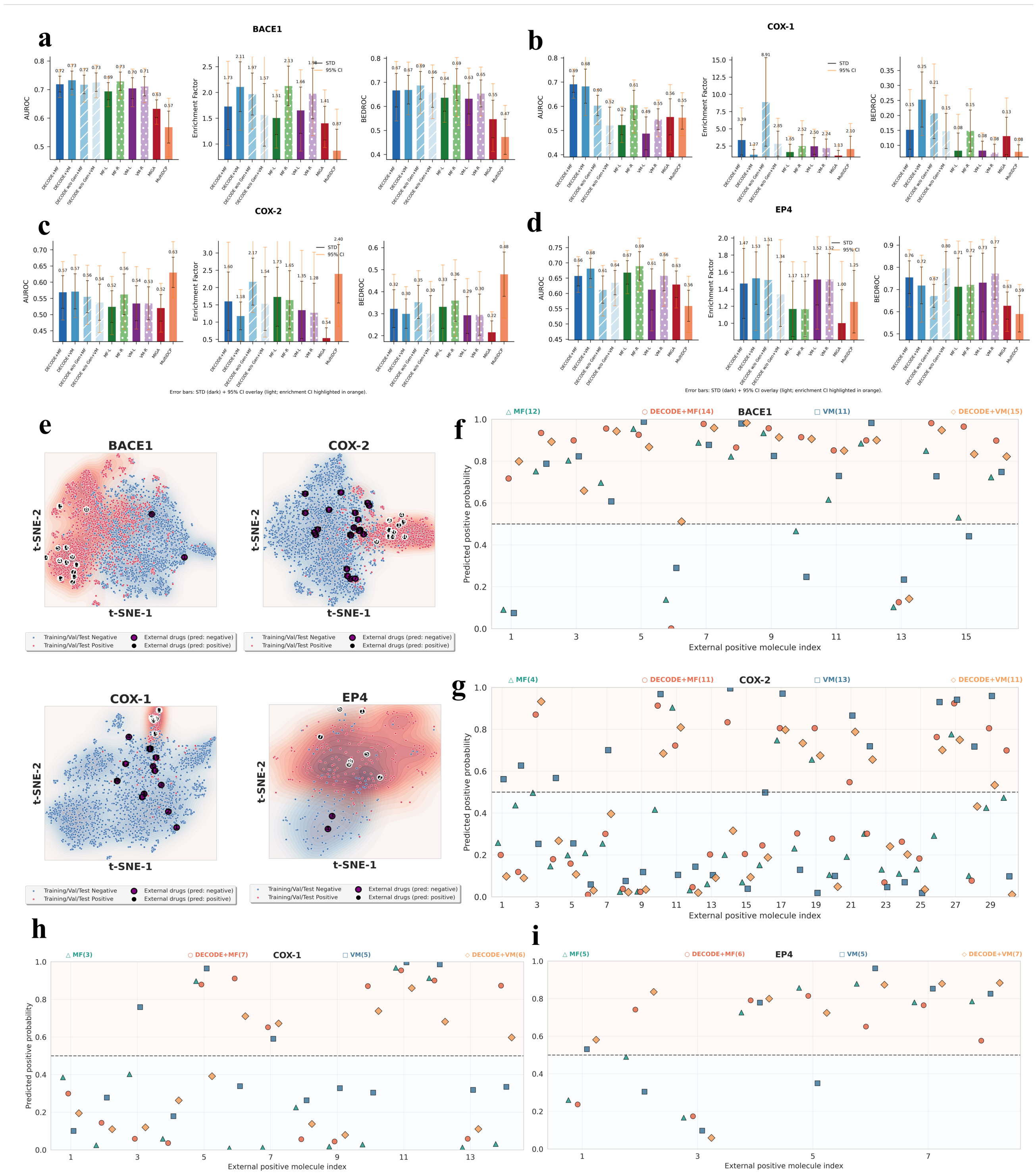

Figure S6: Target-specific ligand screening under UMAP-clustering splits and external known-active recovery. (a–d) Performance on BACE1, COX-1, COX-2, and EP4, evaluated by AUROC, EF1%, and BEDROC20. Dark error bars denote standard deviation, and light orange overlays denote 95% confidence intervals. (e) t-SNE visualization of learned representations for the four target-specific datasets. Training and test compounds are shown together with external known active compounds. (f–i) Predicted positive probabilities for external known active compounds in BACE1, COX-2, COX-1, and EP4. The dashed line denotes the decision threshold. Since these external sets contain known positives only, they are used as recovery analyses rather than full binary screening benchmarks.

S6 Supplementary Evaluation for Downstream Virtual Screening

This section provides detailed downstream screening results supporting the main text Figure 5. We report both UMAP-clustering and Bemis–Murcko scaffold splits, together with external known-active recovery analyses [11; 3; 4]. All enrichment factors are EF1%, and all BEDROC values are BEDROC20 [16].

S6.1 Target-specific ligand screening under UMAP-clustering splits

We first evaluated four target-specific binary screening tasks, including BACE1, COX-1, COX-2, and EP4, under UMAP-clustering splits. This split partitions compounds according to broader chemical neighborhoods and therefore provides a complementary chemically informed evaluation to scaffold splitting [11; 4]. For each target, we report AUROC, EF1%, and BEDROC20 to measure both global discrimination and early recognition [16].

As shown in Supplementary Figure S6, DECODE variants achieve competitive performance across the four targets and often show stronger early-recognition behavior than their corresponding molecular backbones. The external known active compounds are further projected into the learned representation spaces and scored by each model. Since these external compounds contain positives only and do not include naturally matched negatives, they are used only for known-active recovery visualization rather than as full binary screening benchmarks.

S6.2 Cancer activity screening and external known-active recovery

Supplementary Figure S7 provides additional analyses for PRISM anti-cancer activity screening and the MCELC pathway dataset. The dataset-level Sankey diagrams summarize the composition of the PRISM anti-cancer activity task and the MCELC pathway annotations. For PRISM anticancer activity prediction, we report scaffold-split performance using AUROC, EF1%, and BEDROC20, computed on binary test sets containing explicit active and inactive compounds.

We also visualize external anti-cancer known-active recovery using predicted probabilities and t-SNE projection. These external compounds are standardized at the parent-molecule level and deduplicated before evaluation [12; 13; 14; 15]. Because the external list contains only known active compounds, it is not used to compute AUROC, false-positive rate, BEDROC, or enrichment factors. Instead, it is reported as a supporting recovery analysis that examines whether the trained models assign high activity scores to curated known positives.

Finally, Supplementary Figure S7(f) summarizes performance across binary screening tasks under scaffold splits. Scores are normalized by min–max scaling within each task–split and each metric before aggregation. This summary is intended to compare relative model behavior across heterogeneous screening tasks rather than to replace the full task-specific metrics.

S6.3 Target-specific ligand screening under scaffold splits

We further evaluated BACE1, COX-1, COX-2, and EP4 using scaffold splits, which group compounds by their core structure, ensuring no exact scaffold overlap between training and test sets [3]. This approach is commonly used to test how well models generalize to new chemicals. Supplementary Figure S8 shows AUROC, EF1%, and BEDROC20 for all four targets.

In these benchmarks, scaffold splits are not always more difficult than UMAP-clustering splits. Although scaffold splitting removes shared Bemis–Murcko frameworks, it can still preserve local chemical-neighborhood structure or target-specific activity regularities [4]. By contrast, UMAP-clustering splits the partition into broader chemical neighborhoods and produced a more challenging evaluation in our experiments. The relatively larger gains of DECODE under UMAP-clustering splits suggest that biological profile supervision is particularly useful when local chemical-neighborhood cues are less directly

available. We therefore report both split types and interpret them as complementary evaluations rather than ranking one split as universally harder than the other.

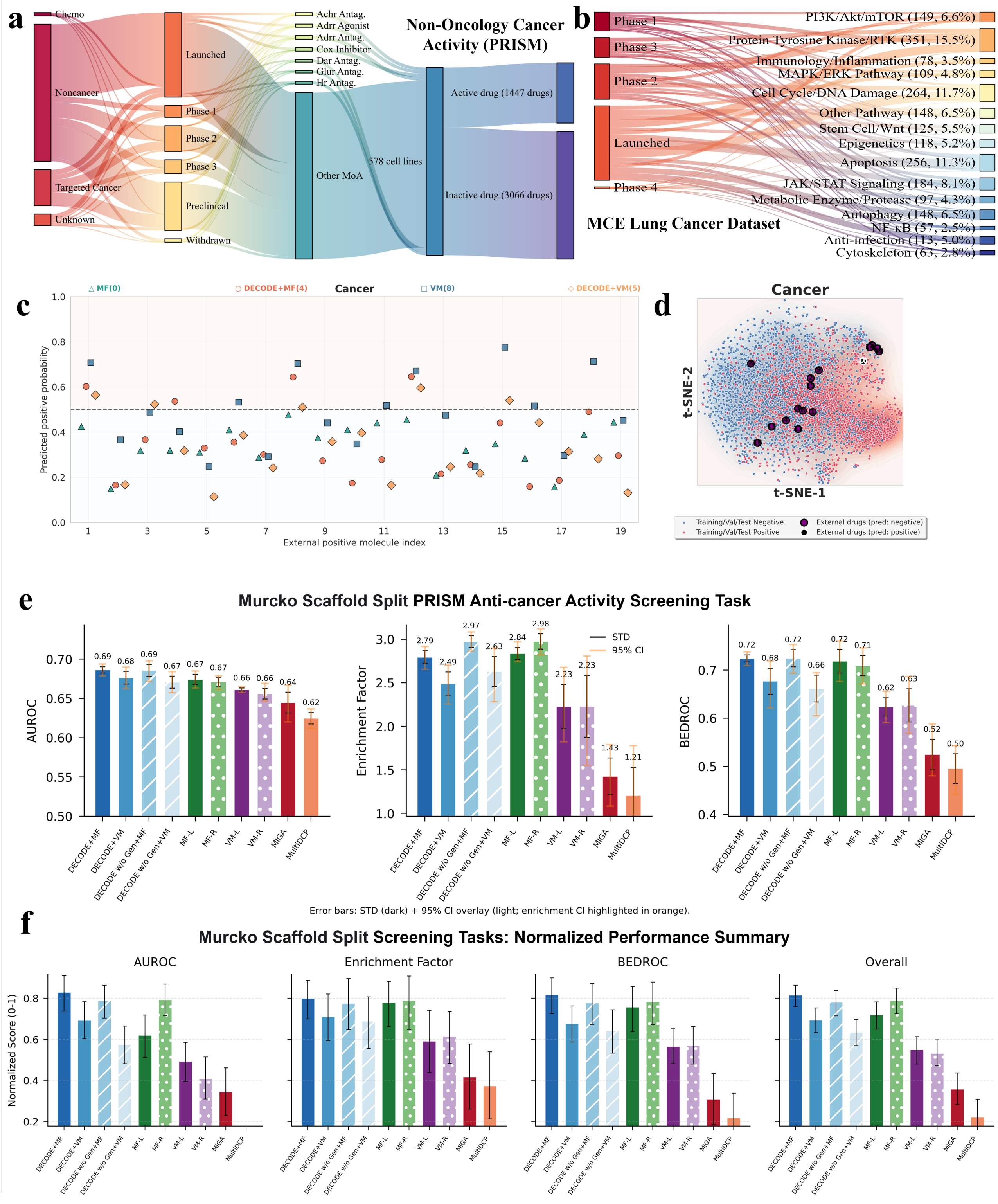

Figure S7: Cancer-related downstream screening and external known-active recovery. (a) Composition of the PRISM anti-cancer activity dataset, including drug categories, clinical stages, annotated mechanisms, and active/inactive labels. (b) Composition of the MCELC pathway dataset, showing clinical stages and pathway annotations. (c) Predicted positive probabilities for external anti-cancer known active compounds. The dashed line denotes the decision threshold. (d) t-SNE visualization of PRISM anti-cancer activity representations, with external known active compounds projected into the learned space. (e) PRISM anti-cancer activity screening under Bemis–Murcko scaffold split, evaluated by AUROC, EF1%, and BEDROC20. (f) Normalized performance summary across binary screening tasks under scaffold splits. Scores are min–max normalized within each task–split and each metric before aggregation. External known-active compounds are used only for recovery visualization and are not treated as full binary screening benchmarks.

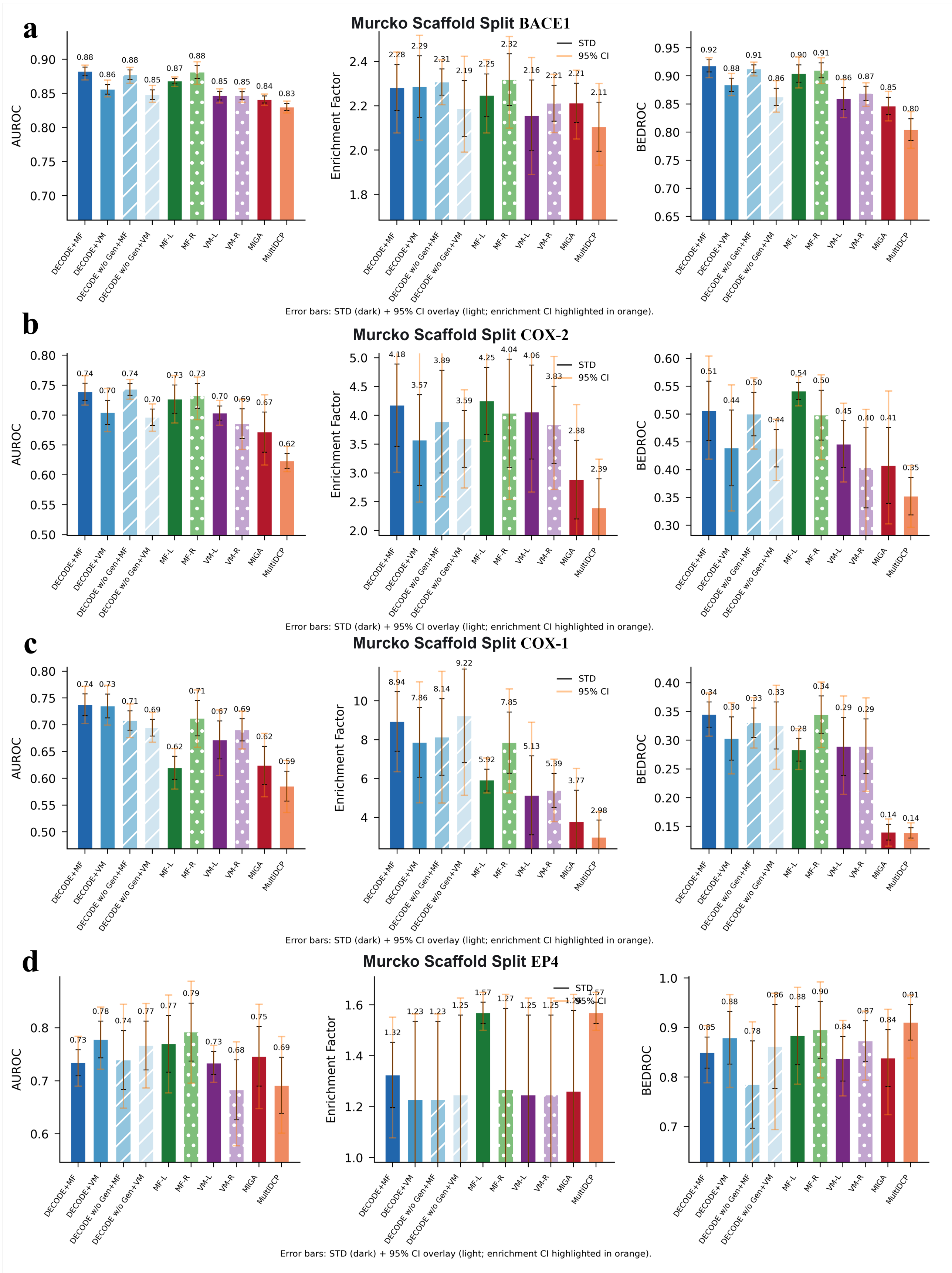

Figure S8: Target-specific ligand screening under Bemis–Murcko scaffold splits. Performance is reported for BACE1, COX-2, COX-1, and EP4 using AUROC, EF1%, and BEDROC20. The scaffold split removes Bemis–Murcko scaffold overlap between training and test compounds and provides a chemically informed evaluation complementary to UMAP-clustering splits. Dark error bars denote standard deviation, and light orange overlays denote 95% confidence intervals.

S7 Ablation and Sensitivity Analyses

This section provides additional ablation studies for DECODE. We examine three questions: whether each architectural component contributes to the learned representation, whether Protocol III gains can be explained by auxiliary reconstruction pretraining alone, and whether the results depend on a specific dose assumption.

Table S1: **Component ablation on LINCS and CDRP.** DECODE and its ablated variants are evaluated under complete-data, morphology-missing, gene-expression-missing, and dual-profile-missing settings. The evaluated components include modal alignment, contrastive consensus, and orthogonal disentanglement, which respectively introduce biological-profile supervision, align shared chemical–biological components, and separate shared response-related information from modality-specific variation. MoA prediction is evaluated by macro F1-score, precision, and recall, while reconstruction is evaluated by Pearson correlation and R^2. The dual-profile missing setting denotes chemical-input inference without measured transcriptomic or morphological profiles. Overall, removing these components generally weakens the primary prediction metrics, supporting their contribution to modality-flexible representation learning.

Dataset: CDRP										
**Inference Scenario**	**Complete Data**			**Morphology Missing**			**Morphology Missing**		**Gene Missing**	
**Model Variant**	F1-Macro	Prec	Rec	F1-Macro	Prec	Rec	Morphology r	Morphology R^2	Gene r	Gene R^2
**DECODE (Full)**	**0.642**±0.070	**0.661**±0.072	0.638±0.067	**0.619**±0.076	**0.632**±0.080	**0.623**±0.073	0.461±0.023	0.107±0.027	0.228±0.017	0.050±0.009
w/o Contrastive Consensus	0.638±0.065	0.657±0.065	0.636±0.066	0.611±0.066	0.619±0.067	0.623±0.067	0.453±0.021	0.109±0.018	0.229±0.020	0.051±0.011
w/o Orthogonal Disentangle.	0.645±0.073	0.664±0.074	**0.643**±0.072	0.608±0.066	0.619±0.068	0.616±0.064	**0.465**±0.022	0.108±0.021	0.227±0.024	0.049±0.011
w/o Modal Alignment	0.589±0.075	0.597±0.077	0.600±0.073	0.574±0.067	0.579±0.070	0.590±0.066	0.463±0.020	0.103±0.026	**0.234**±0.018	**0.053**±0.010
w/o Contrastive + Orth.	0.637±0.082	0.657±0.085	0.635±0.078	0.613±0.078	0.625±0.078	0.619±0.078	0.452±0.022	0.108±0.026	0.233±0.018	0.053±0.009
w/o Contrastive + Align.	0.605±0.093	0.627±0.095	0.605±0.092	0.593±0.068	0.601±0.069	0.606±0.066	0.453±0.023	**0.116**±0.028	0.225±0.022	0.049±0.010
w/o Orth. + Align.	0.625±0.072	0.636±0.076	0.630±0.067	0.594±0.070	0.599±0.074	0.606±0.064	0.460±0.022	0.096±0.031	0.231±0.015	0.051±0.008
w/o All Components	0.624±0.078	0.644±0.082	0.622±0.075	0.597±0.067	0.603±0.072	0.608±0.062	0.454±0.023	0.111±0.025	0.227±0.021	0.050±0.011
Joint Learning (No Align.)	0.555±0.107	0.587±0.104	0.543±0.109	0.625±0.067	0.637±0.071	0.627±0.063	0.461±0.023	0.103±0.025	0.229±0.011	0.050±0.006
Reconstruction Only	-	-	-	-	-	-	0.463±0.023	0.104±0.029	0.224±0.020	0.047±0.010
**Inference Scenario**	**Gene Missing**			**Both Missing (Structure Only)**						
**DECODE (Full)**	**0.616**±0.064	**0.629**±0.064	**0.620**±0.065	**0.583**±0.069	**0.584**±0.070	**0.602**±0.066	0.415±0.024	**0.012**±0.025	0.264±0.023	0.057±0.015
w/o Contrastive Consensus	0.613±0.067	0.623±0.066	0.621±0.068	0.529±0.063	0.523±0.064	0.568±0.058	0.414±0.023	-0.001±0.016	0.259±0.022	0.049±0.015
w/o Orthogonal Disentangle.	0.608±0.073	0.620±0.075	0.613±0.069	0.553±0.065	0.552±0.068	0.584±0.059	**0.417**±0.025	0.018±0.020	0.266±0.029	0.061±0.017
w/o Modal Alignment	0.574±0.083	0.579±0.087	0.592±0.078	0.550±0.067	0.548±0.070	0.581±0.065	0.415±0.023	0.006±0.024	**0.273**±0.020	**0.062**±0.012
w/o Contrastive + Orth.	0.606±0.072	0.617±0.073	0.614±0.072	0.541±0.074	0.537±0.077	0.577±0.067	0.415±0.026	0.004±0.030	0.263±0.016	0.049±0.012
w/o Contrastive + Align.	0.570±0.060	0.577±0.061	0.586±0.062	0.533±0.059	0.529±0.062	0.573±0.055	0.411±0.026	0.009±0.012	0.255±0.025	0.050±0.012
w/o Orth. + Align.	0.596±0.063	0.603±0.064	0.608±0.063	0.558±0.064	0.557±0.069	0.585±0.059	0.416±0.024	0.006±0.028	0.270±0.021	0.060±0.015
w/o All Components	0.592±0.064	0.602±0.066	0.602±0.065	0.546±0.070	0.542±0.074	0.575±0.064	0.413±0.025	0.008±0.018	0.256±0.024	0.050±0.016
Joint Learning (No Align.)	0.576±0.087	0.593±0.083	0.576±0.092	0.585±0.061	0.591±0.064	0.600±0.059	0.414±0.025	0.003±0.034	0.267±0.013	0.059±0.012
Reconstruction Only	-	-	-	-	-	-	0.413±0.026	0.005±0.023	0.263±0.026	0.058±0.016
**Dataset: LINCS**										
**Inference Scenario**	**Complete Data**			**Morphology Missing**			**Morphology Missing**		**Gene Missing**	
**Model Variant**	F1-Macro	Prec	Rec	F1-Macro	Prec	Rec	Morphology r	Morphology R^2	Gene r	Gene R^2
**DECODE (Full)**	**0.908**±0.050	**0.917**±0.045	**0.913**±0.047	**0.906**±0.052	**0.913**±0.049	**0.913**±0.046	0.661±0.030	0.359±0.040	0.402±0.016	0.160±0.013
w/o Contrastive Consensus	0.903±0.056	0.910±0.050	0.911±0.051	0.900±0.058	0.906±0.052	0.910±0.052	0.658±0.027	**0.364**±0.033	0.404±0.015	0.162±0.013
w/o Orthogonal Disentangle.	0.904±0.052	0.912±0.048	0.911±0.046	0.902±0.053	0.907±0.050	0.912±0.047	0.658±0.027	0.351±0.036	0.405±0.016	0.163±0.013
w/o Modal Alignment	0.894±0.055	0.901±0.050	0.903±0.051	0.894±0.054	0.899±0.051	0.905±0.049	0.658±0.027	0.347±0.031	**0.407**±0.015	**0.164**±0.013
w/o Contrastive + Orth.	0.903±0.052	0.911±0.046	0.911±0.047	0.900±0.055	0.907±0.049	0.910±0.050	0.657±0.029	0.359±0.039	0.404±0.017	0.162±0.014
w/o Contrastive + Align.	0.898±0.060	0.904±0.056	0.910±0.052	0.899±0.059	0.904±0.054	0.910±0.051	0.657±0.027	0.363±0.034	0.404±0.015	0.162±0.012
w/o Orth. + Align.	0.892±0.060	0.898±0.057	0.903±0.052	0.894±0.060	0.899±0.057	0.906±0.051	**0.662**±0.027	0.359±0.035	0.403±0.017	0.161±0.014
w/o All Components	0.898±0.054	0.904±0.050	0.910±0.049	0.896±0.051	0.902±0.046	0.908±0.046	0.657±0.031	0.359±0.041	0.404±0.013	0.162±0.011
Joint Learning (No Align.)	0.843±0.041	0.882±0.037	0.837±0.037	0.864±0.043	0.885±0.042	0.868±0.038	0.661±0.025	0.355±0.033	0.405±0.017	0.163±0.014
Reconstruction Only	-	-	-	-	-	-	0.661±0.025	0.355±0.033	0.403±0.016	0.161±0.014
**Inference Scenario**	**Gene Missing**			**Both Missing (Structure Only)**						
**DECODE (Full)**	**0.906**±0.051	**0.914**±0.047	**0.915**±0.046	**0.901**±0.055	**0.907**±0.051	**0.913**±0.047	0.640±0.036	0.276±0.055	0.408±0.020	0.148±0.019
w/o Contrastive Consensus	0.903±0.057	0.908±0.051	0.912±0.052	0.898±0.057	0.904±0.051	0.908±0.052	0.638±0.031	0.257±0.037	0.407±0.019	0.138±0.017
w/o Orthogonal Disentangle.	0.903±0.053	0.909±0.050	0.912±0.046	0.901±0.053	0.907±0.050	0.913±0.047	0.638±0.034	0.266±0.046	**0.412**±0.022	**0.153**±0.019
w/o Modal Alignment	0.895±0.056	0.902±0.052	0.905±0.053	0.896±0.053	0.901±0.050	0.908±0.047	0.637±0.034	0.259±0.041	0.412±0.019	0.150±0.017
w/o Contrastive + Orth.	0.902±0.054	0.910±0.048	0.911±0.049	0.899±0.056	0.906±0.049	0.911±0.050	0.639±0.034	0.253±0.051	0.407±0.021	0.136±0.015
w/o Contrastive + Align.	0.897±0.059	0.902±0.054	0.910±0.050	0.899±0.056	0.906±0.050	0.910±0.049	0.638±0.034	0.260±0.048	0.407±0.018	0.139±0.016
w/o Orth. + Align.	0.896±0.059	0.901±0.055	0.908±0.051	0.892±0.057	0.897±0.054	0.906±0.049	**0.641**±0.034	0.274±0.045	0.408±0.021	0.147±0.018
w/o All Components	0.897±0.051	0.903±0.047	0.909±0.047	0.898±0.049	0.904±0.044	0.911±0.045	0.639±0.036	0.256±0.052	0.408±0.018	0.138±0.014
Joint Learning (No Align.)	0.866±0.046	0.886±0.043	0.869±0.041	0.867±0.057	0.879±0.054	0.881±0.050	0.639±0.032	0.268±0.046	0.410±0.021	0.148±0.018
Reconstruction Only	-	-	-	-	-	-	0.640±0.035	0.275±0.050	0.408±0.018	0.148±0.014

S7.1 Component-level ablation on LINCS and CDRP

We first evaluated the contribution of modal alignment, contrastive consensus, and orthogonal disentanglement on LINCS and CDRP. The ablation variants remove one or more components from the full DECODE framework and are evaluated under different modality-availability settings, including complete data, morphology missing, gene-expression missing, and dual-profile missing.

As shown in Supplementary Table S1, the full DECODE model is generally among the strongest variants and is most consistently competitive, especially under missing-profile settings. Removing modal

alignment reduces the benefit of privileged biological supervision, while removing contrastive consensus weakens the transfer of response-related information into the chemical representation. Removing orthogonal disentanglement also affects performance, supporting the use of geometric separation between shared response-related information and modality-specific variation. However, not every reconstruction-related metric is maximized by the full model; reconstruction is therefore treated as a supporting diagnostic rather than the sole criterion for model selection.

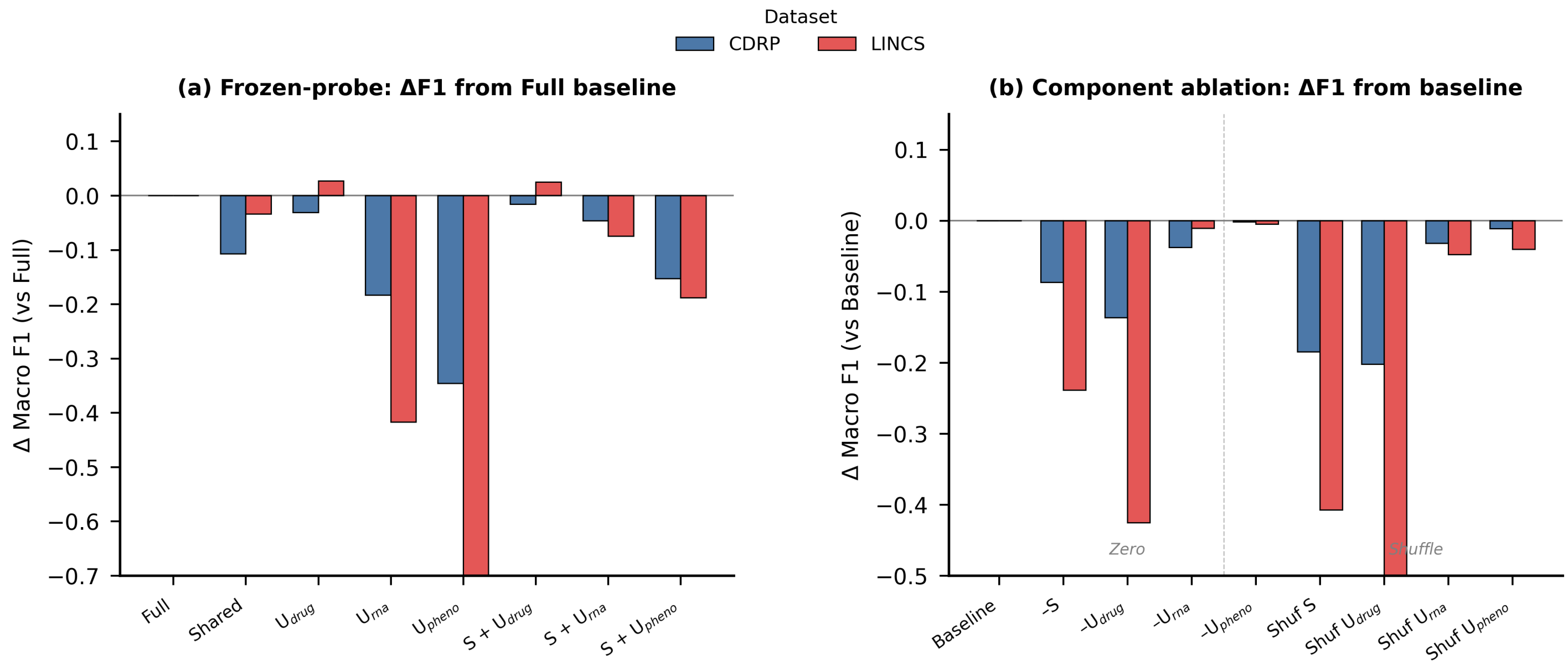

Figure S9: Probing shared and modality-specific components for MoA prediction. (a) Frozen-probe analysis. We freeze the pretrained DECODE encoder and train lightweight classifiers on different subsets of the representation. We report performance as the change in Macro F1 compared to the full representation. Values near zero mean similar performance to the full representation. (b) Component perturbation analysis. We zero or shuffle each component in the full representation before prediction. Zeroing removes the component. Shuffling keeps the marginal distribution but breaks sample-specific information. Negative values mean a lower Macro F1 than the full representation.

S7.2 Probing shared and modality-specific components

To further examine whether the modality-specific components are merely discarded noise, we performed a probing analysis on the pretrained DECODE representations. We first froze the encoder and trained lightweight classifiers on different subsets of the representation. As shown in Supplementary Figure S9(a), the drug-, RNA-, and morphology-specific components all retain measurable MoA-predictive information. The drug-specific component is particularly informative. Shared plus unique components are generally closer to the full representation. This indicates that the full representation benefits from both shared and modality-specific information.

Building on this, we next perturbed the full representation by zeroing or shuffling each component. Supplementary Figure S9(b) shows that disrupting the shared component or the drug-specific component causes the largest performance drop, whereas RNA- and morphology-specific components have smaller but non-zero effects. These results suggest that DECODE does not simply discard all unique information. Instead, orthogonal disentanglement separates shared response-related signals from modality-specific variation, while preserving useful task-relevant information in the unique components.

S7.3 Auxiliary reconstruction control in cancer activity screening

To test whether the improvement of Protocol III is caused simply by auxiliary reconstruction pretraining or a more expressive downstream predictor, we compared DECODE with a drug-only auxiliary-reconstruction initialization, denoted as AuxRecon-init. This control uses molecular inputs and the same

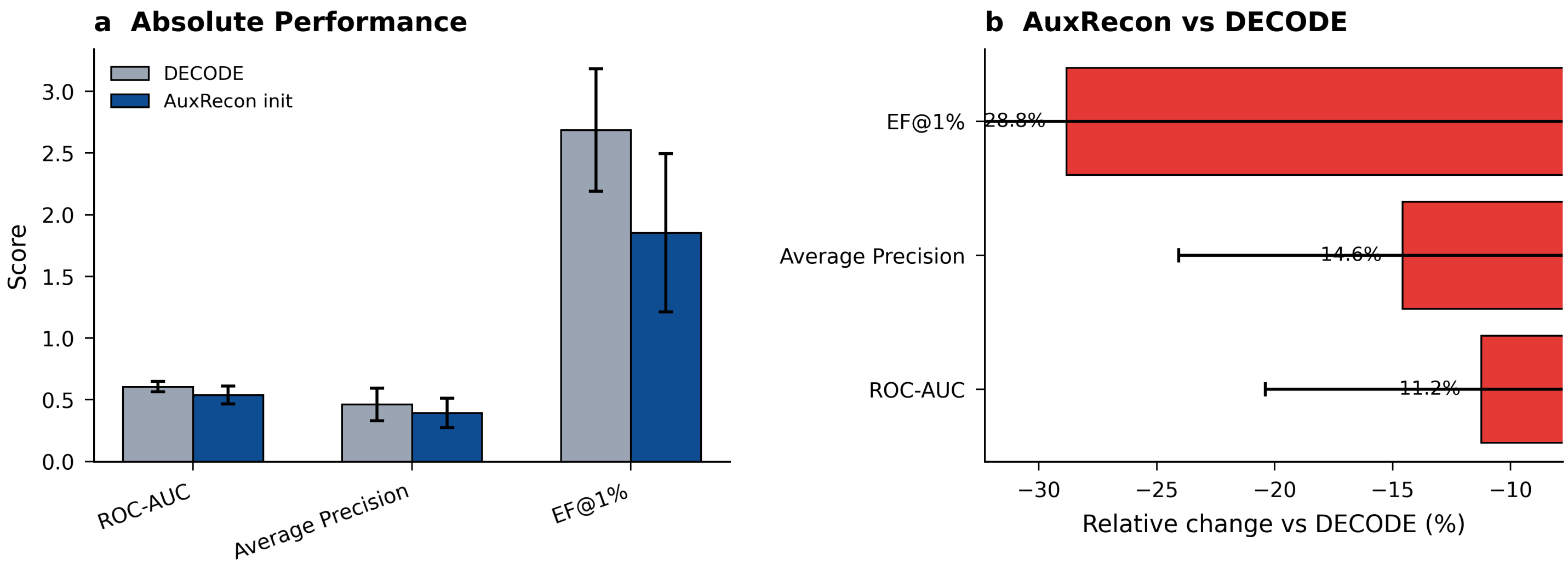

Figure S10: Auxiliary reconstruction control for cancer activity screening. (a) Absolute performance of DECODE and the drug-only auxiliary-reconstruction initialization (AuxRecon-init) on ROC-AUC, average precision, and EF1%. (b) Relative change of AuxRecon-init compared with DECODE. Negative values indicate lower performance than DECODE. AuxRecon-init shows relative decreases of 11.2% in ROC-AUC, 14.6% in average precision, and 28.8% in EF1%, suggesting that the gain of DECODE is not explained by auxiliary reconstruction pretraining alone.

downstream cancer activity screening setting, but does not use paired transcriptomic and morphological alignment or geometric disentanglement.

Supplementary Figure S10 shows numerically higher ROC-AUC, average precision, and EF1% for DECODE than for AuxRecon-init. AuxRecon-init shows relative decreases of 11.2% in ROC-AUC, 14.6% in average precision, and 28.8% in EF1% compared with DECODE. The larger descriptive gap on EF1% suggests that biological-profile supervision may be helpful for early active-compound prioritization, which is central to virtual screening.

S7.4 Dose sensitivity analysis

Because DECODE includes a dose-aware structural encoder, we checked if the reported performance depends on a particular dose setting. In LINCS-like data, the available concentrations are discrete experimental conditions, and DECODE is not intended to learn a continuous pharmacological dose-response function from these sparse anchors. The dose context is therefore interpreted as an experimental-context variable used by the gate. Specifically, we tested: (1) fixed-dose anchors (0.1, 1.0, 5.0, and 10.0 μM), where the dose value is predetermined; (2) marginalized predictions, where outputs are averaged over multiple fixed anchors; (3) a learnable scalar dose, which is initialized at 5.0 μM and then trained on the training split as a task-adaptive context; (4) random dose sampling, where the dose is randomly chosen during training; and (5) a no-dose variant, where the dose gate is bypassed entirely.

As shown in Supplementary Figure S11, ROC-AUC and average precision on cancer activity prediction vary only slightly across dose configurations. Held-out retrieval Recall@10 and mAP on LINCS and CDRP are also stable, with changes close to zero for most variants. These results indicate that DECODE is weakly sensitive to inference-time dose assumptions. Importantly, the no-dose variant remains comparable to dose-conditioned variants, suggesting that biological-profile-free inference is not driven by post-hoc dose selection or memorization of a specific discrete concentration anchor.

Table S2: Comparative data distribution for the Non-Oncology Cancer Activity [5] and MedChemExpress Lung Cancer datasets. Activity Classification and MOA Prediction tasks originate from the Non-Oncology Cancer Activity corpus, with MOA categories containing fewer than ten samples discarded. Pathway Prediction is based on the MedChemExpress Lung Cancer dataset after excluding pathway labels represented by fewer than two observations. The imbalance ratio is defined as the ratio between the minimum and maximum class counts.

	**Activity Classification**	**MOA Prediction**	**Pathway Prediction**
Dataset	Non-Oncology Cancer Activity	Non-Oncology Cancer Activity	MedChemExpress Lung Cancer
#Train	2900	1534	475
#Val	622	329	102
#Test	622	329	102
Imbalance ratio	0.4869	0.1149	0.0192
Number of Classes	2	93	94

Table S3: Statistical overview of datasets for four common drug targets from the ChEMBL database. This table summarizes the datasets for beta-secretase 1 (BACE1), cyclooxygenase-1 (COX-1), cyclooxygenase-2 (COX-2), and prostaglandin E receptor 4 (EP4). The data is partitioned into training (#Train), validation (#Val), and test (#Test) sets. The IC50 threshold (in nM) defines the cutoff for classifying compounds as active (positive) or inactive (negative). The ratio of positive to negative samples (#Pos/#Neg) and the imbalance ratio (calculated as #Neg/#Pos) quantify the class distribution. All datasets are configured for a single binary classification task (#Tasks), with the Receiver Operating Characteristic Area Under the Curve (ROC-AUC) as the evaluation metric.

	**BACE1**	**COX-1**	**COX-2**	**EP4**
#Train	4802	2396	3491	245
#Val	1029	513	748	52
#Test	1029	514	749	53
IC50 thresh (nM)	100	100	100	100
#Pos/#Neg	2563/4297	142/3281	1006/3982	133/217
Imbalanced ratio	0.597	0.043	0.253	0.613
#Tasks	1			
Metric	ROC-AUC			

Table S4: Curated external validation set for Cancer (19 compounds). All SMILES were standardized to canonical form using RDKit, with salt and counterion removal, parent-compound normalization, and InChIKey-based deduplication.

**Idx**	**Name**	**SMILES**
1	mevastatin	CC[C@H](C)C(=O)O[C@H]1CCC=C2C=C[C@H](C)[C@H](CC[C@@H]3C[C@@H](O)CC(=O)O3)[C@H]21
2	disulfiram	CCN(CC)C(=S)SSC(=S)N(CC)CC
3	gestrinone	C#C[C@]1(O)CC[C@H]2[C@@H]3CCC4=CC(=O)CCC4=C3C=C[C@@]21CC
4	zardaverine	COc1cc(-c2ccc(=O)[nH]n2)ccc1OC(F)F
5	DCEBIO	CCn1c(O)nc2cc(Cl)c(Cl)cc21
6	drospirenone	C[C@]12CCC(=O)C=C1[C@@H]1C[C@@H]1[C@H]1[C@@H]3[C@@H]4C[C@@H]4[C@@]4(CCC(=O)O4)[C@@]3(C)CC[C@@H]12
7	anagrelide	O=C1CN2Cc3c(ccc(Cl)c3Cl)N=C2N1
8	lovastatin	CC[C@H](C)C(=O)O[C@H]1C[C@@H](C)C=C2C=C[C@H](C)[C@H](CC[C@@H]3C[C@@H](O)CC(=O)O3)[C@H]21
9	danazol	C#C[C@]1(O)CCC2C3CCC4=Cc5oncc5C[C@]4(C)C3CC[C@@]21C
10	tanaproget	Cn1c(C#N)ccc1-c1ccc2c(c1)C(C)(C)OC(=S)N2
11	P22077	CC(=O)c1cc([N+](=O)[O-])c(Sc2ccc(F)cc2F)s1
12	simvastatin	CCC(C)(C)C(=O)O[C@H]1C[C@@H](C)C=C2C=C[C@H](C)[C@H](CC[C@@H]3C[C@@H](O)CC(=O)O3)[C@H]21
13	benzethonium	CC(C)(C)CC(C)(C)c1ccc(OCCOCC[N+](C)(C)Cc2ccccc2)cc1
14	tioxolone	O=c1oc2cc(O)ccc2s1
15	pitavastatin	O=C(O)C[C@H](O)C[C@H](O)/C=C/c1c(C2CC2)nc2ccccc2c1-c1ccc(F)cc1
16	norethindrone	C#C[C@]1(O)CC[C@H]2[C@@H]3CCC4=CC(=O)CC[C@@H]4[C@H]3CC[C@@]21C
17	bis(maltolato)oxovanadium(IV)	Cc1occc(=O)c1O
18	tepoxalin	COc1ccc(-n2nc(CCC(=O)N(C)O)cc2-c2ccc(Cl)cc2)cc1
19	PR-619	N#CSc1cc(SC#N)c(N)nc1N

Table S5: Curated external validation set for BACE1 (16 compounds). All SMILES were standardized to canonical form using RDKit, with salt and counterion removal, parent-compound normalization, and InChIKey-based deduplication.

**Idx**	**Name**	**SMILES**
1	BACE1/2-IN-1	C[C@@H]1C[C@H]1COC(C)(C)[C@@H]1C[C@H]1[C@]12CN(c3ncccn3)C[C@H]1CSC(N)=N2
2	NB-360	Cc1cc(C#N)cnc1C(=O)Nc1ccc(F)c([C@]2(C)CO[C@@](C)(C(F)(F)F)C(N)=N2)c1
3	BACE1-IN-1	C[C@]1(c2cc(NC(=O)c3ccc(C#N)cn3)ccc2F)N=C(N)OCC1(F)F
4	BACE1-IN-4	C[C@]1(c2cc(NC(=O)c3cnc(OCF)cn3)ccc2F)N=C(N)SCC12CCS(=O)(=O)CC2
5	AMG-8718	CC1(C#Cc2cnc3c(c2)[C@]2(COC(N)=N2)c2cc(-c4cccnc4F)ccc2O3)COC1
6	AChE/BChE/BACE-1-IN-2	Fc1ccc(CNC2CCN(Cc3ccccc3)C2)c(F)c1
7	AM-6494	C#CCOc1cnc(C(=O)Nc2cc(F)c(F)c([C@@]3(C)N=C(N)S[C@@]4(COC)C[C@H]43)c2)cn1
8	BACE1-IN-13	COc1cnc(C(=O)Nc2ccc(F)c([C@]3(C)Cn4nc(C#N)cc4C(N)=N3)c2)cn1
9	JNJ-67569762	CCS(=O)(=O)[C@]1(C)CC[C@@](CF)(c2cc(NC(=O)c3cc4c(cn3)OC(F)(F)O4)ccc2F)N=C1N
10	LY2886721	NC1=N[C@@]2(c3cc(NC(=O)c4ccc(F)cn4)ccc3F)COC[C@H]2CS1
11	Umibecestat	C[C@@]1(c2nc(NC(=O)c3ncc(C(F)(F)F)cc3Cl)ccc2F)CO[C@@](C)(C(F)(F)F)C(N)=N1
12	BACE-1 inhibitor 2	C[C@H]1C[C@H]2CC(F)(F)C(N)=N[C@@]2(c2cc(NC(=O)c3cnc(OCF)cn3)ccc2F)CO1
13	β-Secretase Inhibitor IV	C[C@@H](NC(=O)c1cc(C(=O)N[C@@H](Cc2ccccc2)[C@H](O)CNC2CC2)cc(N(C)S(C)(=O)=O)c1)c1ccccc1
14	BACE1-IN-6	C[C@]1(c2cc(/C=C(\F)c3ccc(C#N)cn3)ccc2F)N=C(N)S[C@@]2(C(=O)N3CCOCC3)C[C@H]21
15	BACE-1 inhibitor 1	NC1=N[C@@](c2cc(NC(=O)c3ccc(Br)cn3)ccc2F)(C(F)F)COC1
16	BACE1-IN-2	C[C@]1(c2cc(NC(=O)c3ccc(C#N)cn3)ccc2F)N=C(N)CO[C@H]1C(F)(F)F

Table S6: Curated external validation set for COX-1 (14 compounds). All SMILES were standardized to canonical form using RDKit, with salt and counterion removal, parent-compound normalization, and InChIKey-based deduplication.

**Idx**	**Name**	**SMILES**
1	Eltenac	O=C(O)Cc1cscc1Nc1c(Cl)cccc1Cl
2	Indomethacin sodium hydrate	COc1ccc2c(c1)c(CC(=O)OC/C=C(\C)CC/C=C(\C)CCC=C(C)C)c(C)n2C(=O)c1ccc(Cl)cc1
3	Indomethacin	COc1ccc2c(c1)c(CC(=O)O)c(C)n2C(=O)c1ccc(Cl)cc1
4	Diclofenac	O=C(O)Cc1ccccc1Nc1c(Cl)cccc1Cl
5	Mofezolac	COc1ccc(-c2noc(CC(=O)O)c2-c2ccc(OC)cc2)cc1
6	S-(+)-Ketoprofen	C[C@H](C(=O)O)c1cccc(C(=O)c2ccccc2)c1
7	Ketoprofen	CC(C(=O)O)c1cccc(C(=O)c2ccccc2)c1
8	Tenidap-d3	NC(=O)N1C(=O)C(C(=O)c2cccs2)c2cc(Cl)ccc21
9	Tenidap	NC(=O)N1C(=O)/C(=C(\O)c2cccs2)c2cc(Cl)ccc21
10	Ketorolac	O=C(c1ccccc1)c1ccc2n1CCC2C(=O)O
11	SC-560	COc1ccc(-n2nc(C(F)(F)F)cc2-c2ccc(Cl)cc2)cc1
12	FR122047	COc1ccc(-c2nc(C(=O)N3CCN(C)CC3)sc2-c2ccc(OC)cc2)cc1
13	Lornoxicam	CN1C(C(=O)Nc2ccccn2)=C(O)c2sc(Cl)cc2S1(=O)=O
14	Bromfenac	Nc1c(CC(=O)O)cccc1C(=O)c1ccc(Br)cc1

Table S7: Curated external validation set for COX-2 (30 compounds). All SMILES were standardized to canonical form using RDKit, with salt and counterion removal, parent-compound normalization, and InChIKey-based deduplication.

Idx	Name	SMILES
1	Eltenac	O=C(O)Cc1cscc1Nc1c(Cl)cccc1Cl
2	DuP-697	CS(=O)(=O)c1ccc(-c2cc(Br)sc2-c2ccc(F)cc2)cc1
3	Imrecoxib	CCCN1CC(c2ccc(S(C)(=O)=O)cc2)=C(c2ccc(C)cc2)C1=O
4	Indomethacin (Indometacin) sodium hydrateis	COc1ccc2c(c1)c(CC(=O)OC/C=C(\C)CC/C=C(\C)CCC=C(C)C)c(C)n2C(=O)c1ccc(Cl)cc1
5	Indomethacin farnesil	COc1ccc2c(c1)c(CC(=O)OCC=C(C)CCC=C(C)CCC=C(C)C)c(C)n2C(=O)c1ccc(Cl)cc1
6	Indomethacin	COc1ccc2c(c1)c(CC(=O)O)c(C)n2C(=O)c1ccc(Cl)cc1
7	Diclofenac	O=C(O)Cc1ccccc1Nc1c(Cl)cccc1Cl
8	S-(+)-Ketoprofen	C[C@H](C(=O)O)c1cccc(C(=O)c2ccccc2)c1
9	Ketoprofen	CC(C(=O)O)c1cccc(C(=O)c2ccccc2)c1
10	COX-2-IN-17	COC(=O)[C@@H](N)Cc1cn(-c2nc(Cl)nc(N3CCCCC3)n2)c2ccccc12
11	SC57666	CS(=O)(=O)c1ccc(C2=C(c3ccc(F)cc3)CCC2)cc1
12	Anti-inflammatory agent 8	Cc1c(C(=O)NNC(=S)Nc2ccccc2)sc2nc3ccccc3n12
13	COX-2-IN-21	COc1cc(C2CC(c3cccc(-n4cnnn4)c3)=NN2C(C)=O)cc(OC)c1OC
14	Thioflosulide	CS(=O)(=O)Nc1cc2c(cc1Sc1ccc(F)cc1F)C(=O)CC2
15	S-2474	CCN1CC/C(=C\c2cc(C(C)(C)C)c(O)c(C(C)(C)C)c2)S1(=O)=O
16	SC-58125	CS(=O)(=O)c1ccc(-n2nc(C(F)(F)F)cc2-c2ccc(F)cc2)cc1
17	COX-2-IN-26	CSc1nc2ccccc2n1-c1csc(NCC(C)=NN=C2SCC(=O)N2c2ccccc2)n1
18	Valdecoxib	Cc1onc(-c2ccccc2)c1-c1ccc(S(N)(=O)=O)cc1
19	Tilmacoxib	Cc1nc(C2CCCCC2)c(-c2ccc(S(N)(=O)=O)c(F)c2)o1
20	Fenofibric acid	CC(C)(Oc1ccc(C(=O)c2ccc(Cl)cc2)cc1)C(=O)O
21	Desmethyl Celecoxib	NS(=O)(=O)c1ccc(-n2nc(C(F)(F)F)cc2-c2ccccc2)cc1
22	Celecoxib	Cc1ccc(-c2cc(C(F)(F)F)nn2-c2ccc(S(N)(=O)=O)cc2)cc1
23	Rofecoxib	CS(=O)(=O)c1ccc(C2=C(c3ccccc3)C(=O)OC2)cc1
24	Lornoxicam	CN1C(C(=O)Nc2ccccn2)=C(O)c2sc(Cl)cc2S1(=O)=O
25	COX-2-IN-20	COC(=O)c1nc(CCl)n(-c2ccc(F)cc2)n1
26	COX-2/5-LOX-IN-2	NS(=O)(=O)c1ccc(-n2nc(C(=O)O)cc2-c2cc3ccccc3s2)cc1
27	COX-2-IN-28	CSc1nc2ccccc2n1-c1csc(NCC(C)=NN=c2scc(-c3ccc(C)cc3)n2-c2ccccc2)n1
28	FR-188582	CS(=O)(=O)c1ccc(-c2cc(Cl)nn2-c2ccccc2)cc1
29	Bromfenac	Nc1c(CC(=O)O)cccc1C(=O)c1ccc(Br)cc1
30	COX-2/15-LOX-IN-1	CSc1nc2ccccc2n1-c1csc(NCC(C)=NNC(=S)Nc2ccccc2)n1

Table S8: Curated external validation set for EP4 (8 compounds). All SMILES were standardized to canonical form using RDKit, with salt and counterion removal, parent-compound normalization, and InChIKey-based deduplication.

Idx	Name	SMILES
1	ER-819762	CCN1C(=O)N2Cc3cc(OC)cc(OC)c3[C@@H](C)C=C2C12CCN(Cc1cc(C)cc(C)c1)CC2
2	BAY-1316957	CCn1c2ccc(C)cc2c2cc(-c3nc4c(C)c(C(=O)O)ccc4n3CCOC)ccc21
3	Grapiprant	CCc1nc2c(C)nc(C)cc2n1-c1ccc(CCNC(=O)NS(=O)(=O)c2ccc(C)cc2)cc1
4	Palupiprant	C[C@H](NC(=O)c1c(C(F)F)nn(C)c1Oc1cccc(C(F)(F)F)c1)c1ccc(C(=O)O)cc1
5	CJ-42794	C[C@H](NC(=O)c1cc(Cl)ccc1Oc1ccc(F)cc1)c1ccc(C(=O)O)cc1
6	MK-2894	Cc1sc(C)c(C(=O)NC2(c3ccc(C(=O)O)cc3)CC2)c1Cc1ccc(C(F)(F)F)cc1
7	TP-16	C[C@H](NC(=O)c1c(Cc2ccc(F)cc2)sc2c1CCOC2)c1ccc(C(=O)O)cc1
8	EP4 receptor antagonist 1	C/C=C/c1nnn(Cc2ccc(C(F)(F)F)cc2)c1C(=O)N[C@@H](C)c1ccc(C(=O)O)cc1

Table S9: **Summary of baseline models and adaptation rules.** The table summarizes the baseline families used in the revised evaluation, their input modalities, and their role in controlling different sources of performance gain. For published baselines, we followed the released implementations and original training designs as closely as possible, with necessary adaptations limited to dataset formatting, task-specific output heads, and modality-availability settings. No DECODE-specific modules were added to the baseline models.

**Baseline family**	**Representative models**	**Input modality at inference**	**Purpose in comparison**	**Implementation / adaptation**
Chemical-only backbones	MolFormer [18], VideoMol [9]	Molecular structure only	Evaluate strong molecular representations without biological-profile supervision.	Trained under the same downstream split, validation, and metric protocol as DECODE.
Capacity-controlled chemical baselines	MolFormer-LargeHead, VideoMol-LargeHead	Molecular structure only	Test whether performance gains are due to a larger prediction head rather than biological-profile supervision.	The molecular backbone is kept the same, while the classifier capacity is increased to match the downstream predictor size.
Single-modality biological baselines	Gene-only, morphology-only	Measured transcriptomic or morphological profiles	Evaluate the predictive signal of each biological modality alone.	Used only when the corresponding measured biological profile is available.
Fusion baselines	Early fusion, late fusion	Available chemical and/or biological modalities	Test whether direct combination of raw modalities is sufficient.	Early fusion concatenates input features directly; late fusion encodes each modality separately before concatenation.
Recent multimodal / response-prediction baselines	MIGA [19], MultiDCP [20]	Inputs required by the original method and the evaluated missing-modality setting	Compare DECODE with recent multimodal alignment or drug-response prediction methods.	Followed released implementations and original training designs as closely as possible. Adaptations were limited to dataset interfaces, task-specific output heads, and modality-availability settings.
DECODE variants	DECODE(MF), DECODE(VM), ablated variants	Molecular structure, optionally with available biological profiles depending on protocol	Evaluate the contribution of biological-profile supervision, backbone choice, and geometric disentanglement.	DECODE(MF) uses MolFormer embeddings; DECODE(VM) uses VideoMol embeddings. Ablated variants remove specific DECODE components.

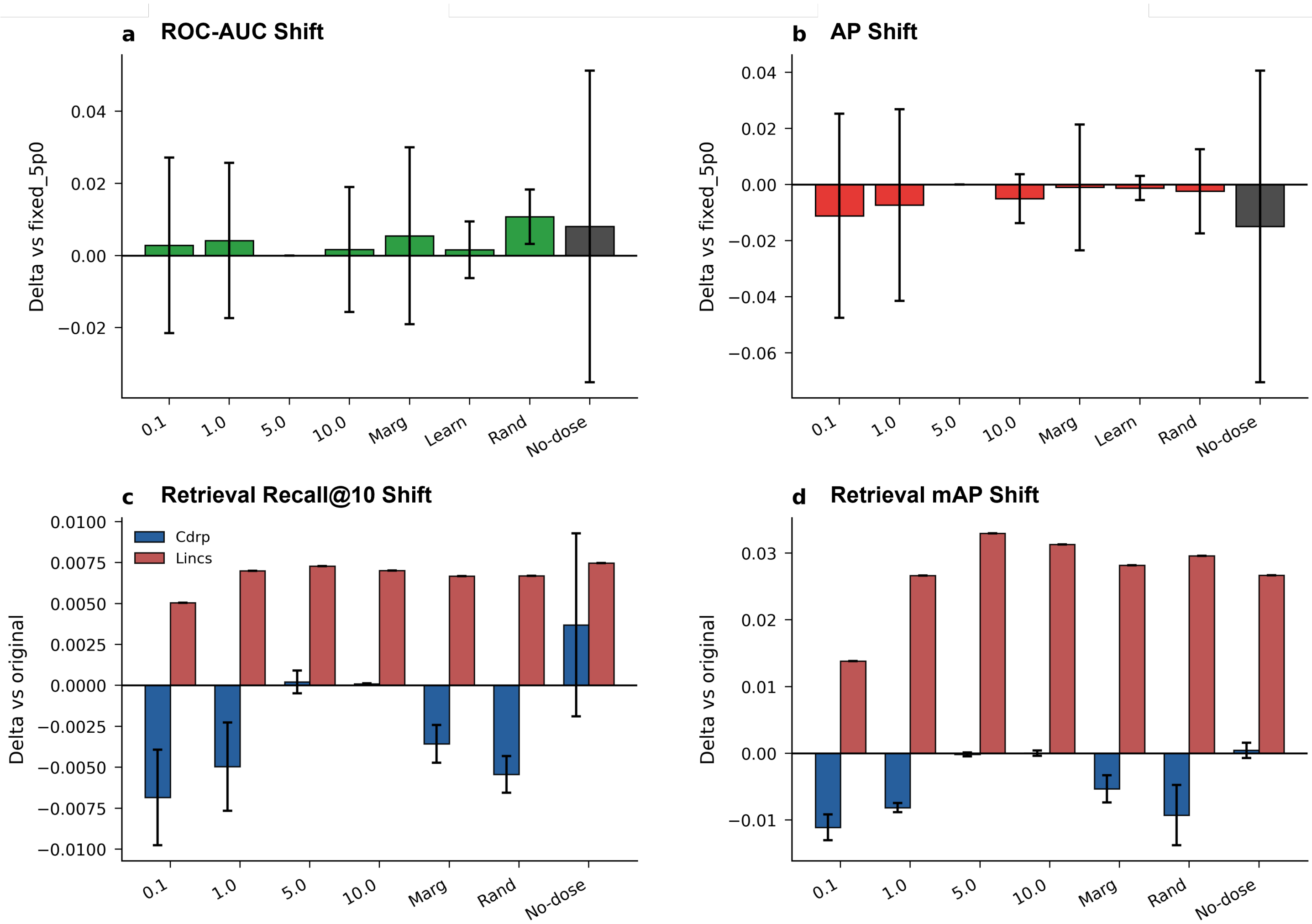

Figure S11: Dose sensitivity analysis. (a,b) Changes in ROC-AUC and average precision on cancer activity prediction under different dose settings, relative to the fixed 5.0 μM reference. (c,d) Changes in held-out retrieval Recall@10 and mAP on CDRP and LINCS under different dose settings, relative to the original setting. Evaluated variants include fixed dose anchors (0.1, 1.0, 5.0, and 10.0 μM), marginalized dose prediction, learnable dose, random dose sampling, and a no-dose variant that bypasses the dose gate. The small shifts across variants suggest that DECODE does not rely heavily on specific inference-time dose assumptions.

References

[1] T. Natoli, G. Way, X. Lu, B. Cimini, D. Logan, K. Karhohs, J. Caicedo, M. Alimova, K. Hartland, T. Golub, et al., *Version v1 (Zenodo)* **2021**, *10*.

[2] M.-A. Bray, S. M. Gustafsdottir, M. H. Rohban, S. Singh, V. Ljosa, K. L. Sokolnicki, J. A. Bittker, N. E. Bodycombe, V. Dančík, T. P. Hasaka, et al., *Gigascience* **2017**, *6*, 12 giw014.

[3] G. W. Bemis, M. A. Murcko, *Journal of Medicinal Chemistry* **1996**, *39*, 15 2887.

[4] Q. Guo, S. Hernandez-Hernandez, P. J. Ballester, *Journal of Cheminformatics* **2025**, *17* 94.

[5] S. M. Corsello, R. T. Nagari, R. D. Spangler, J. Rossen, M. Kocak, J. G. Bryan, R. Humeidi, D. Peck, X. Wu, A. A. Tang, et al., *Nature cancer* **2020**, *1*, 2 235.

[6] A. González, A. Covarrubias-Pinto, R. M. Bhaskara, M. Glogger, S. K. Kuncha, A. Xavier, E. Seemann, M. Misra, M. E. Hoffmann, B. Bräuning, et al., *Nature* **2023**, *618*, 7964 394.

[7] M. Fidelle, C. Rauber, C. Alves Costa Silva, A.-L. Tian, I. Lahmar, A.-L. M. de La Varende, L. Zhao, C. Thelemaque, I. Lebhar, M. Messaoudene, et al., *Science* **2023**, *380*, 6649 eabo2296.

[8] J. D. Minna, J. A. Roth, A. F. Gazdar, *Cancer cell* **2002**, *1*, 1 49.

[9] H. Xiang, L. Zeng, L. Hou, K. Li, Z. Fu, Y. Qiu, R. Nussinov, J. Hu, M. Rosen-Zvi, X. Zeng, et al., *Nature Communications* **2024**, *15*, 1 9696.

[10] A. Gaulton, A. Hersey, M. Nowotka, A. P. Bento, J. Chambers, D. Mendez, P. Mutowo, F. Atkinson, L. J. Bellis, E. Cibrián-Uhalte, et al., *Nucleic acids research* **2017**, *45*, D1 D945.

[11] L. McInnes, J. Healy, N. Saul, L. Grossberger, *Journal of Open Source Software* **2018**, *3*, 29 861.

[12] D. Fourches, E. Muratov, A. Tropsha, *Journal of Chemical Information and Modeling* **2010**, *50*, 7 1189.

[13] D. Fourches, E. Muratov, A. Tropsha, *Journal of Chemical Information and Modeling* **2016**, *56*, 7 1243.

[14] A. P. Bento, A. Hersey, E. Félix, G. Landrum, A. Gaulton, F. Atkinson, L. J. Bellis, M. De Veij, A. R. Leach, *Journal of Cheminformatics* **2020**, *12* 51.

[15] S. R. Heller, A. McNaught, I. Pletnev, S. Stein, D. Tchekhovskoi, *Journal of Cheminformatics* **2015**, *7* 23.

[16] J.-F. Truchon, C. I. Bayly, *Journal of Chemical Information and Modeling* **2007**, *47*, 2 488.

[17] A. v. d. Oord, Y. Li, O. Vinyals, *arXiv preprint arXiv:1807.03748* **2018**.

[18] J. Ross, B. Belgodere, V. Chenthamarakshan, I. Padhi, Y. Mroueh, P. Das, *Nature Machine Intelligence* **2022**, *4*, 12 1256.

[19] S. Zheng, J. Rao, J. Zhang, L. Zhou, J. Xie, E. Cohen, et al., *Advanced Science* **2024**, *11*, 32 2404845.

[20] Y. Wu, Q. Liu, Y. Qiu, L. Xie, *PLOS Computational Biology* **2022**, *18*, 8 e1010367.
